# A Synaptic Mechanism for Temporal Filtering of Visual Signals

**DOI:** 10.1371/journal.pbio.1001972

**Published:** 2014-10-21

**Authors:** Tom Baden, Anton Nikolaev, Federico Esposti, Elena Dreosti, Benjamin Odermatt, Leon Lagnado

**Affiliations:** 1 MRC Laboratory of Molecular Biology, Neurobiology Division, Cambridge, United Kingdom; 2 School of Life Sciences, University of Sussex, Brighton, United Kingdom; Stanford University School of Medicine, United States of America

## Abstract

The visual system transmits information about fast and slow changes in light intensity through separate neural pathways. We used *in vivo* imaging to investigate how bipolar cells transmit these signals to the inner retina. We found that the volume of the synaptic terminal is an intrinsic property that contributes to different temporal filters. Individual cells transmit through multiple terminals varying in size, but smaller terminals generate faster and larger calcium transients to trigger vesicle release with higher initial gain, followed by more profound adaptation. Smaller terminals transmitted higher stimulus frequencies more effectively. Modeling global calcium dynamics triggering vesicle release indicated that variations in the volume of presynaptic compartments contribute directly to all these differences in response dynamics. These results indicate how one neuron can transmit different temporal components in the visual signal through synaptic terminals of varying geometries with different adaptational properties.

## Abstract 2

Synaptic volume matters! The size of the presynaptic compartment of retinal bipolar cells controls the amplitude, speed, and adaptation of synaptic transmission.

## Author Summary

The process of neurotransmission involves the conversion of electrical signals into the release of a chemical neurotransmitter from the neurons synaptic terminal, and the key trigger for this release is a rise in calcium concentration. Accordingly, the amplitude and speed of this calcium signal controls the amplitude and time-course of synaptic communication. Working on the synaptic terminals of fish retinal bipolar cells, we show that the presynaptic calcium signal and the subsequent neurotransmitter release are shaped by the basic property of synapse volume. Using a combination of experimental approaches and computational models, we found that large synapses are slow and adapt little during ongoing stimulation, while small synapses are fast and show more profound adaptation. This observation leads to a second key concept: since neurons usually have several presynaptic terminals that may vary in volume, a single neuron can, in principle, forward different synaptic signals to different postsynaptic partners. We provide direct evidence that this is the case for bipolar cells of the fish retina.

## Introduction

The retina analyzes the visual world through a series of spatio-temporal filters that establish parallel representations for transmission to the brain [1]–[4]. The anatomical organization of these channels is established in the inner plexiform layer (IPL), which is organized into five to six distinct strata containing the dendrites of as many as 20 different types of retinal ganglion cell (RGC) [2]. In each stratum, bipolar cells (BCs) with distinct filtering properties make excitatory synaptic connections with defined subsets of RGCs [5]. For instance, “transient” RGCs are thought to receive excitatory inputs from BCs with bandpass characteristics, while “sustained” RGCs receive inputs from BCs acting as low-pass filters [6].

A long-standing question is how multiple spatio-temporal filters are built using the limited numbers of neurons responding to a particular region of the visual field [7]–[9]. An answer might lie in reconsidering the fundamental neural element through which the visual signal is transmitted to the inner retina: although individual BCs have diverse properties, their output is transmitted through a much more numerous and heterogeneous component of neural circuits–synapses [10]. Indeed, recent evidence indicates that different synapses of the same BC transmit the visual signal with varying kinetics because of local interactions with different types of inhibitory amacrine cells [11]. Here we ask whether heterogeneous transmission of the visual signal might also reflect intrinsic variations between the various synaptic compartments of a BC. The property we concentrate on is the volume of the terminal, which is expected to influence the amplitude and kinetics of the presynaptic calcium signal controlling neurotransmission [12].

To observe the activity of multiple BC synapses we used zebrafish expressing fluorescent proteins reporting the fusion of synaptic vesicles or the presynaptic calcium signal driving fusion [13],[14]. Here we demonstrate that BC terminals of different sizes tend to transform the visual signal in different ways. On average, smaller terminals generate calcium transients that are larger and faster, resulting in a higher initial gain of responses to an increase in temporal contrast followed by more profound adaptation. Small terminals also transmit high frequencies more effectively. Such differences in the outputs of small and large terminals are also observed in individual cells, which therefore have an intrinsic ability to filter visual information through channels with different gains, temporal filters and adaptive properties.

## Results

### The Volume of Bipolar Cell Terminals Determines the Gain and Kinetics of Signal Transmission

**Figure 1:**
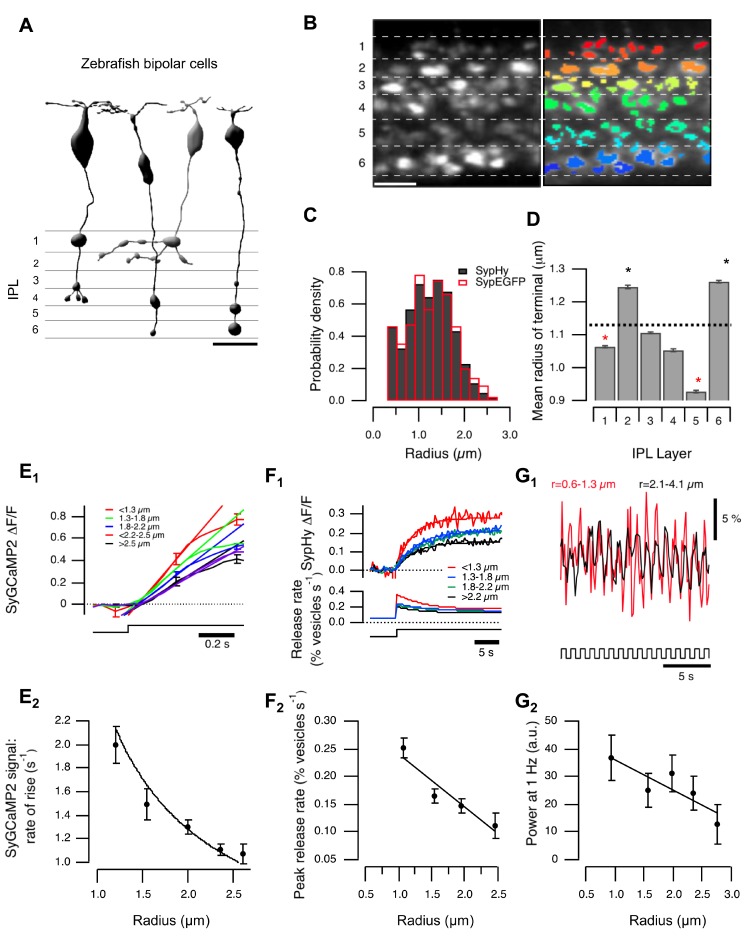
Variations in terminal size, calcium signals, and vesicle release. (A) BCs transmit through multiple terminals. Left: published examples of zebrafish cone BCs [64] illustrate the range of presynaptic terminal sizes. Scalebar = 10 µm. (B) BC terminals in the IPL of a zebrafish (10 dpf) expressing sypHy under control of the *ribeye* promoter. Left: Raw image showing six strata of the IPL. Right: Overlay of ROIs defining terminals. Scale bar = 10 µm. (C) The distribution of the effective terminal radii. Black bars show estimates obtained in fish expressing sypHy (*n* = 5,061 terminals from seven fish), and the red bars shows the distribution measured in fish expressing synaptophysin-EGFP (*n* = 421 terminals from one fish). Distribution of sizes calculated with SyGCaMP2 is shown in Figure S1H. (D) Variations in average radius of terminals in each stratum. The average radius over the whole IPL was 1.13±0.40 µm (dashed line). Stars mark strata in which the average radius was significantly greater or smaller (*p*<0.001, Wilcoxon rank-sum test, *n* varies between 560 and 930 terminals). Similar distributions calculated for individual layers are shown in Figure S1I. (E) Relation between terminal size and calcium signals. (E_1_) SyGCaMP2 signals in response to a step of light (λ = 590 nm; for details see methods) in ON terminals, averaged over groups of the effective radius shown (*n* = 143, 347, 286, 114, and 36, respectively). Straight lines fitted over the initial phase. Significant difference between groups 1, 3, and 5 (Student's *t* test: *p*_(1–3)_ = 0.004; *p*_(3–5)_ = 0.005; *p*_(1–5)_<0.001). (E_2_) The rate of rise of the SyGCaMP2 signal varies as the inverse of the radius, as shown by the fitted curve (*n* = 926 from five fish). Spearman correlation coefficient = −1, critical value (*p* = 0.05) = 0.85. (F) Relation between terminal size and peak rate of vesicle release. (F_1_) SypHy signals in response to a step of light in ON terminals, averaged over groups of the effective radius shown (*n* = 117, 176, 113, and 33 terminals from five fish). The lower panel shows the conversion into relative release rates, as described [14]. (F_2_) The initial rate of vesicle release as a function of radius (total *n* = 438 terminals from five fish). The points fall on a line. Spearman correlation = −1, critical value (*p* = 0.05) = 0.9. (G) terminal size and modulation of presynaptic calcium. (G_1_) SyGCaMP2 signal driven by modulation of light intensity at 1 Hz (100% contrast, square wave). Red trace averaged over OFF contrast responding terminals with r = 0.6–1.3 µm; black trace averaged over OFF contrast responding terminals with r = 2.1–4.1 µm. (G_2_) relative power of the signal at 1 Hz as a function of radius (*n* = 98, 89, 68, 37, and 9 terminals from four fish). Points were fitted with a line. Responses to light decrements are shown in Figure S1G.

In the vertebrate retina, signals from rod and cone photoreceptors travel through BCs to be transmitted through synaptic terminals of various sizes (Figure 1A). To survey the activity of many BC synapses in a live animal, we imaged eight to ten days post fertilisation (dpf) zebrafish larvae expressing sypHy, a reporter of vesicle fusion (Figure 1B) [14],[15], or SyGCaMP2, which signals the presynaptic calcium transient [13],[16]. Regions of interest defining individual terminals in images of the IPL were generated using an algorithm based on a Laplace operator (Figure 1B) [17], from which we estimated the “effective terminal radius” (Materials and Methods). A survey of 5,061 terminals revealed a wide variation in radius with a mean of 1.13±0.40 µm (Figure 1C, black bars). Terminals of different sizes were not distributed randomly through the IPL (Figures 1D and S1I): terminals in layers 2 and 6 were significantly larger than the average across the whole population of BCs, while terminals in layers 1, 3, 4, and 5 were significantly smaller.

How do these variations in the volume of the presynaptic compartment affect the calcium signal that is generated when a stimulus alters the membrane potential and the rate of calcium influx? To investigate this question we imaged SyGCaMP2 and measured the average response over the whole region of interest (ROI) defining each terminal. Although this procedure will neglect calcium gradients within the terminal, these gradients will not be maintained when calcium influx is modulated at frequencies relevant to vision (see below). In a sample of 932 ON terminals, the initial rate of rise of the calcium signal elicited by a full field step of light was approximately twice as fast in the smallest terminals compared to the largest, and the calcium concentration reached a higher steady-state level (Figure 1E_1,2_). Variations in terminal size were also correlated with differences in the kinetics of exocytosis, as measured in fish expressing sypHy. In a sample of 438 ON terminals, the relative rate of vesicle release in response to the same stimulus (quantified as a percentage of vesicles released per second) varied by a factor of at least two between the smallest and largest terminals (Figure 1F_1,2_) [14],[18]. A similar relationship between release amplitude and terminal size was observed in the OFF channel (Figure S1G).

A correlation between the size of a terminal and the amplitude of its response was also observed when the temporal contrast of the stimulus was increased while keeping the mean luminance constant. Figure 1G_1_ shows averaged SyGCaMP2 responses to modulation of light intensity at 1 Hz (100% contrast). The power of the calcium signal at the fundamental frequency was greater in small terminals (r = 0.6–1.3 µm) than in large (r = 2.1–4.1 µm), varying by a factor of at least three across the population (Figure 1G_2_).

**Figure 2:**
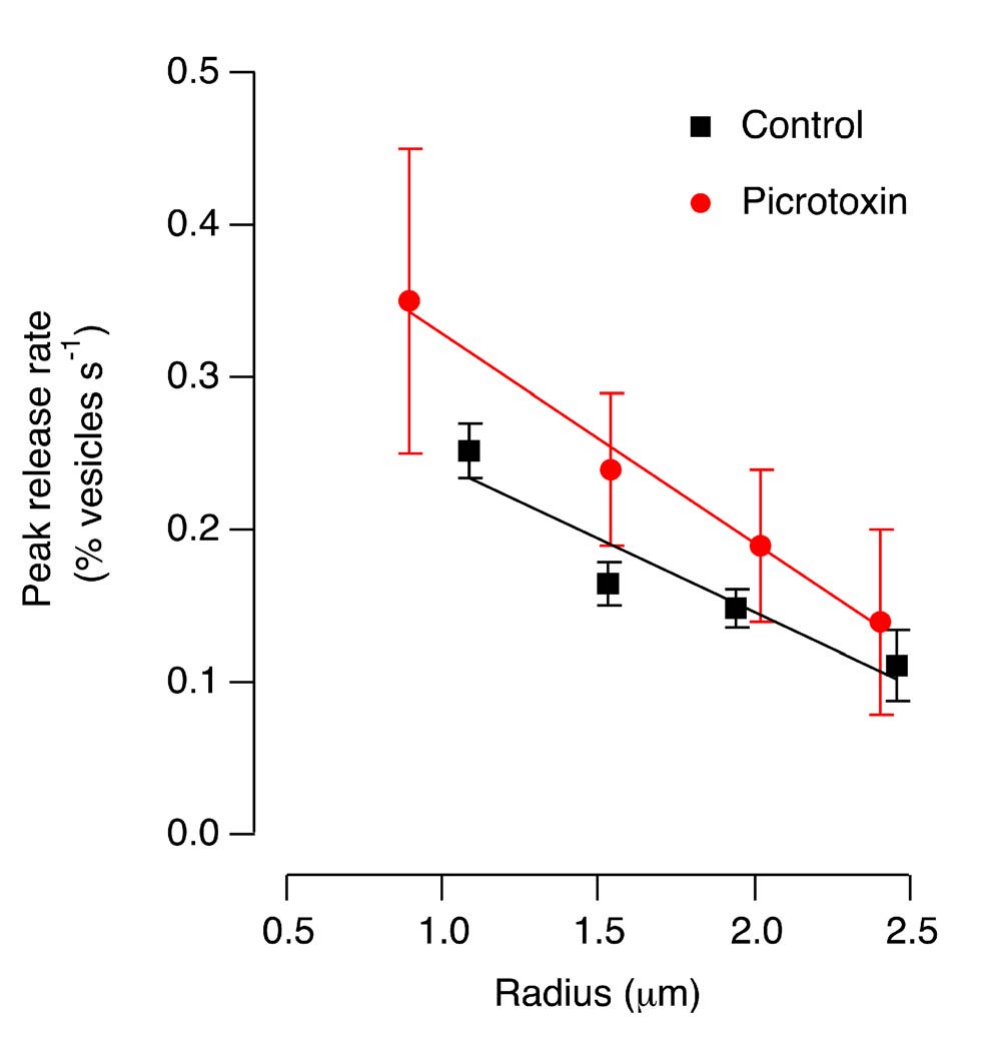
The relation between terminal volume and release rate persists when GABAergic feedback inhibition is blocked. The initial rate of release in response to a step of light in control terminals (black, from Figure 1F_2_) is compared with measurements in which 100 M picrotoxin was injected in the front of the eye (77 ON terminals from three fish). The line fitted to the control has a slope of −0.096±0.023, and the line fitted to the picrotoxin measurements has a slope of −0.138±0.01 dependence peak release size (Figure 1F_2_). Note that picrotoxin also increased the amplitude of the exocytic response, as would be expected when feedback inhibition is reduced.

Does the correlation between the size of the terminal compartment and the release rate reflect the network in which the terminal is embedded? For instance, one scenario might be that large terminals respond with lower gain because they receive stronger inhibitory feedback from amacrine cells. To test this possibility, we blocked GABAergic inhibition through ionotropic receptors by injecting 100 µM picrotoxin into the eye (Figure 2). This manipulation increased the amplitude of the exocytic response across terminals of all sizes, as would be expected when feedback inhibition is reduced [18],[19], but larger terminals continued to respond with lower gain. This observation suggests that the correlation between terminal size and release rate reflects intrinsic properties of the terminal rather than the influence of the network it is embedded in.

Might the correlation between stimulus-dependent changes in the brightness of terminals and estimates of their size be caused by a bias in the detection algorithm, perhaps causing smaller terminals to be detected only when their fluorescence signal was strong enough? Two lines of evidence indicated that this was not the case. First, the distribution of terminal sizes was very similar when measured in transgenic fish expressing synaptophysin-enhanced green fluorescent protein (EGFP), a fluorescent marker of synapses that is not affected by neural activity (Figure 1C, red bars). Second, estimates of terminal size did not differ significantly when measurements were compared under conditions of high and low activity, as described in Figure S1 (See Methods). The results in Figure 1 therefore indicate that the volume of the presynaptic compartment is closely linked to the gain with which BCs transmit the visual signal to the inner retina.

### Different Calcium Dynamics in Small and Large Terminals of Individual Bipolar Cells

**Figure 3:**
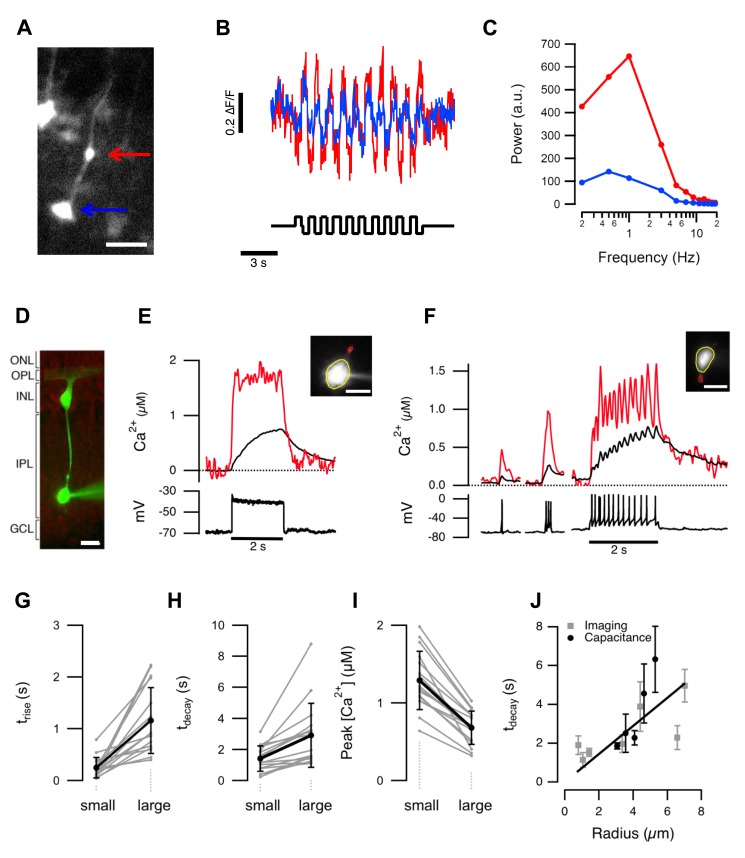
Different calcium signals in small and large terminals of the same cell. (A) A BC expressing GCaMP5. Large and small terminals are indicated by the blue and red arrows. Scalebar 10 µm. (B) Responses of the terminals in (A) to a stimulus modulated at 1 Hz. (C) Power spectrum of the responses from large and small terminals calculated for different stimulus frequencies. (D) Mixed BC filled with OGB-5N in a slice of goldfish retina. Scale bar 10 µm. (E) Spatially averaged Ca^2+^ signals in the small (red) and large (black) terminal during a 2 s depolarizing current step (10 pA). This BC did not generate spikes. (F) A comparison of Ca^2+^ signals in the small and large terminal of a “spiking” BC. Each spike caused a calcium transient that was larger and faster in the smaller terminal. (G–I) Time constants of the calcium signal rise (t_rise_), decay (t_decay_), and peak amplitude, evaluated in 16 pairs of small and large terminals. For small and large, τ_rise_ = 0.25±0.20 and 1.16±0.64 s; τ_decay_ = 1.42±0.82 and 2.91±2.05 s; amplitude = 1.29±0.38 and 0.68±0.21 µM. All these parameters were significantly different in small and large terminals (*p*<0.001; Wilcoxon ranked sum, *n* = 16 cells from nine adult retinae). The average radii of the small and large terminals were 1.1±0.3 and 5.2±1.2 µm, respectively. (J) Time constants of calcium decay were directly proportional to terminal radius determined using imaging (grey, *n* = 32 terminals) or capacitance measurements (black, *n* = 20 terminals). The linear fit was constrained to go through the origin. Error bars show 1 standard deviation (SD).

Are differences in gain maintained in small and large terminals of the same cell? We investigated this question both *in vivo* and *in vitro*. *In vivo* experiments used zebrafish transiently expressing Ribeye::GCaMP5 in sparse populations of BCs. Square-wave stimuli (100% contrast) were presented at various frequencies, and Figure 3A–3C shows results from one BC responding to a 1 Hz stimulus. The calcium signal in the smaller of the two terminals clearly exhibited the larger degree of modulation (Figure 3B; cf. Figure 1G_1_), and the power of the response peaked at a higher frequency (Figure 3C). Qualitatively similar results were obtained from four BCs.

We next used slices of goldfish retina to make whole-cell recordings directly from the large terminal of “mixed” BCs, which is connected to smaller presynaptic compartments by narrow processes. Introduction of the Ca^2+^ indicator Oregon green BAPTA (OGB-5N) allowed us to compare presynaptic calcium signals in the large and small compartments while achieving direct electrical control of the terminal system (Figure 3D; cf. Figure 1A) [20]. A BC generating a graded voltage response is shown in Figure 3E. The OGB-5N signal in the smaller terminal (red) rose faster than that in the large (black), reached more than double the concentration, and then decayed more rapidly. Similar results were observed in eight cells generating purely graded responses.

Many BCs in the retina of fish generate voltage spikes as well as graded responses [16],[21],[22], and terminal volume also affected the presynaptic calcium transient generated by these electrical responses [16],[21]–[23]. In the example in Figure 3F, the small terminal (red) responded to each spike with a clear Ca^2+^ transient, but the signal in the large terminal (black) was smaller and slower, and the response to a train of spikes was dominated by the gradual accumulation of Ca^2+^. Figures 3G–3I summarize these properties in 16 pairs of terminals (eight graded and eight spiking), each pair from a different BC. In all cases, global Ca^2+^ signals were faster and larger in the smaller of two connected terminals. The same observation was made when the patch-pipette was placed on the soma (Figure S3). The results in Figure 3 demonstrate that variations in terminal volume profoundly affected the activity of different synapses providing the output from a single BC.

### The Relation between the Decay of the Calcium Transient and the Radius of the Terminal

The time-constant of decay of a calcium transient (τ_decay_) was directly proportional to the radius of the terminal (r), as shown Figure 3J. This observation was made both when r was estimated from images of terminals filled with dye, and when r was calculated from the capacitance of terminals detached from the cell body. This simple linear relation is notable because it is predicted by single compartment models of calcium dynamics in which there are no significant calcium gradients [24],[25]. The appropriateness of a single compartment model for BC terminals can be understood in terms of the characteristic time, t_eq_, with which a calcium gradient collapses in a volume of radius r after calcium influx ceases:
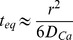
where D_Ca_ is the diffusion coefficient of calcium [26]. Assuming D_Ca_ = 220 µm^2^/s [27], t_eq_, is only ∼0.75 ms in a terminal of r = 1 µm, indicating that a calcium gradient will collapse on a much shorter time-scale than stimuli relevant to vision. Even in the largest terminals with r = 5 µm, t_eq_ is ∼19 ms, which is less than half the period of a stimulus fluctuating at 20 Hz. The lack of any appreciable calcium gradients on these time-scales was confirmed in a 3-D model in which calcium influx occurred through clusters of calcium channels (Figure S3). Calcium signals on these spatial scales have been demonstrated to trigger neurotransmitter release from ribbon synapses of fish bipolar cells, although calcium nanodomains very close to calcium channels also play a role in ribbon-type synapses in other neurons and other species [28].

These considerations indicate that there is a direct and causal link between the volume of the presynaptic compartment and the amplitude and kinetics of the calcium transient caused by a stimulus. But why is the volume of the presynaptic compartment also correlated with its output measured as vesicle release (Figure 1F)? Such a link is expected, because it is the calcium signal in the terminal that drives the output.

### A Single-Compartment Model of Presynaptic Calcium Dynamics

**Figure 4:**
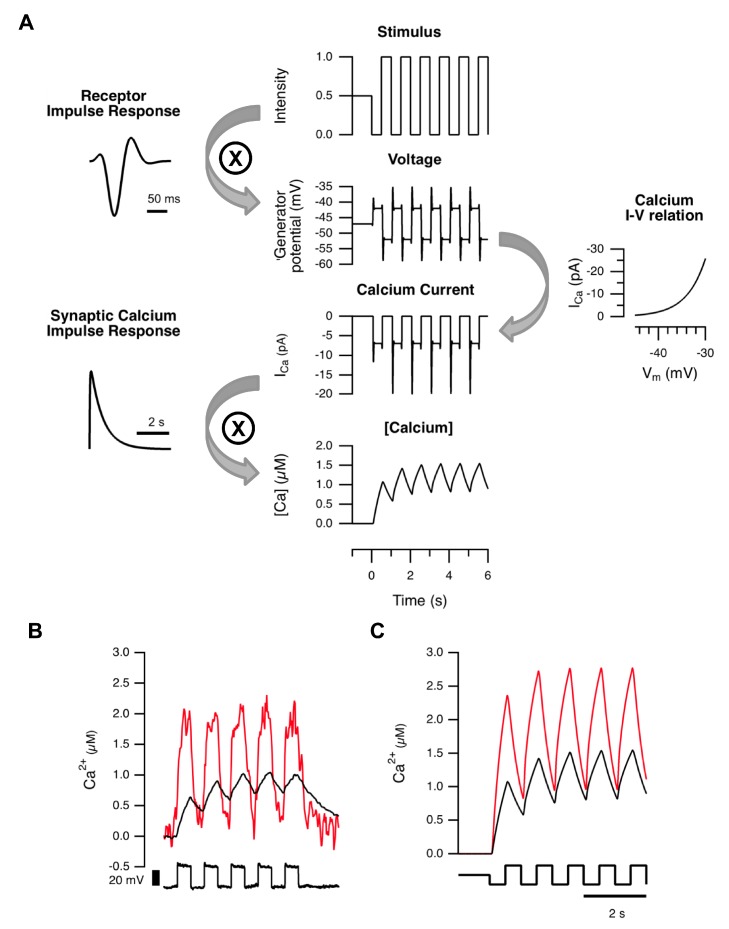
A simple model to predict global calcium changes in the presynaptic terminal. (A) The stimulus (here a 1 Hz square wave) was convolved with the photoreceptor impulse response to estimate membrane voltage (top). Subsequently, the current through L-type calcium channels was calculated based on the I–V relation and number of channels (middle). Convolution of the calcium current with the synaptic calcium impulse response, calculated from Figure 3G, yielded an estimate of global calcium concentration over time (bottom). (B, C) Measured (B), and modeled (C), global calcium changes in a small (red; radius = 1 µm) and large (black; radius = 3 µm) terminals responding to a 1 Hz square wave stimulus. Data in (B) from goldfish “mixed” BC (cf. Figure 3D–3J).

**Figure 5:**
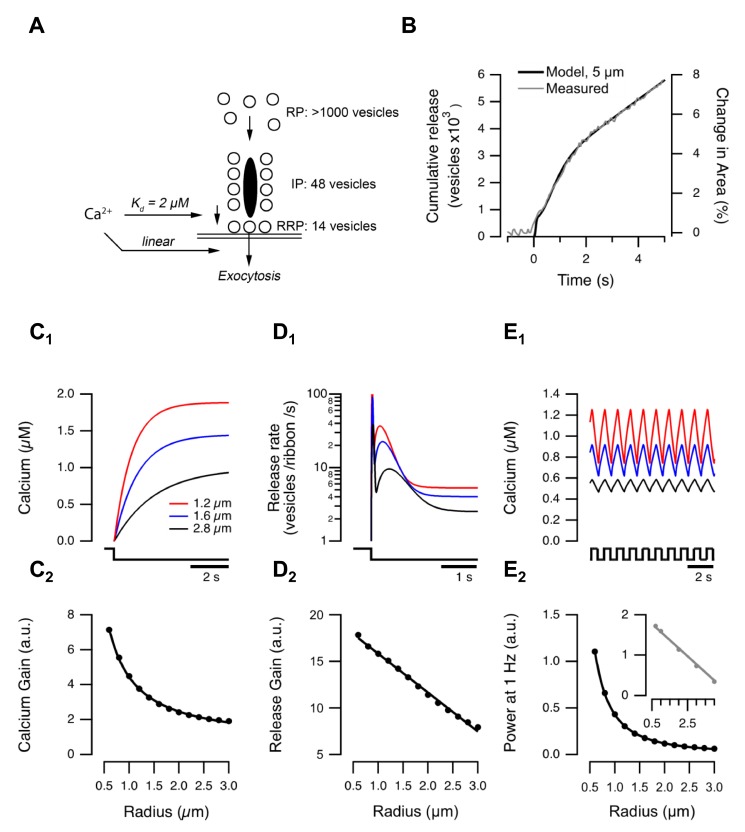
A model of vesicle release through the ribbon. (A) Schematic showing three vesicle pools involved in the exocytic response triggered by calcium. (B) The model (black) closely reproduces the three phases of release measured by [45] (grey) upon maximal activation of calcium channels. (C_1_) Bulk calcium and (D_1_) vesicle release modeled for OFF terminals with different radii (1.2, 1.6, 2.8 µm) when a light step is turned off. (C_2_) The initial rate of rise of the calcium signal (calcium gain) varies as 1/r, while release gain falls linearly with r (D_2_). (E_1_) Calcium in response to a 1 Hz square wave stimulus in different size terminals. (E_2_) Power at the stimulus frequency falls with 1/r (cf. Figure 1E_2_). See also Figure S4. (E_2_) Inset: unlike the one-compartment model, the 3-D diffusion model (Figure S3) predicts a linear relation between power at the stimulus frequency and radius.

To explore how variations in the size of BC terminals might impact on transmission of visual signals we modeled this process in two stages, described in detail in Text S1. The output from the first stage was the dynamics of calcium in the terminal, and is shown schematically in Figure 4A. The second stage used these calcium dynamics to predict the kinetics of vesicle release, as described in Figure 5B and below.

To model the presynaptic calcium signal, the light stimulus was convolved with the impulse response of a BC to predict the voltage trajectory in the soma (Figure 4A, top). BCs in goldfish have bandpass characteristics with peak transmission varying between about 1–14 Hz [22], and we chose a value of 9.5 Hz [29]. The voltage response in the soma was then instantaneously mapped to the presynaptic calcium current using the I–V relation and density of L-type calcium channels measured in the terminal of BCs isolated from goldfish [30]. This I–V relation is an exponential function, changing e-fold in ∼6.6 mV over the physiological range of membrane potentials [30]. These first two steps in the model are analogous to the linear-nonlinear (LN) model that has been used to provide a description of responses in RGCs [7]. This model therefore identifies the rectifying I–V relation of the calcium current in BCs as the first major non-linearity in retinal processing. The time-course of the presynaptic calcium signal in response to a visual stimulus was calculated by convolving the time-course of calcium influx with the “presynaptic calcium filter” predicted by the one-compartment model, which has an impulse response decaying with time-constant τ_decay_ (Figure 3E and 3F). The value of τ_decay_ depended on the radius of the terminal, according to the linear relation measured experimentally in Figure 3J.

To test this model of calcium dynamics we compared its predictions with experimental measurements made with OGB-5N in BCs from slices of goldfish retina. The calcium concentration in the smaller of two connected synaptic compartments rose higher and faster, and then decayed almost to baseline after each cycle of a 1 Hz stimulus, while in the larger terminals there was an accumulation of calcium and much smaller modulations in concentration (Figure 4B). The model could account for these differences by using the appropriate value of r while keeping all other variables constant (Figure 4C).

We considered two extensions of this model. The first is the use of three dimensions to estimate changes in calcium concentration at the active zone: this provided a very similar prediction to the one-compartment model (Figure S3). The second extension was to add active conductances in the terminal leading to calcium spikes, but again this did not alter the main conclusions that we could draw about the impact of terminal volume on gain and kinetics (see below and Figure S6).

### A Model of Synaptic Transmission through Bipolar Cells

Having established that the model accounted adequately for variations in the presynaptic calcium signal, we extended it to explore the impact of terminal size on the kinetics of vesicle release. The following properties of exocytosis at the ribbon synapse of BCs were taken into account, all of which have been measured experimentally.

#### Property 1

There are three anatomically distinct populations of vesicles in the synaptic terminal of BCs: the rapidly-releasable pool (RRP) docked at the active zone, the intermediate pool (IP) attached to the ribbon behind the active zone, and the reserve pool (RP) that is mobile across the whole terminal [31]. Based on the assumption of a constant ribbon density (see below), the sizes of the RRP and IP were proportional to terminal surface area, while RP size depended on volume (equation 4 in Text S1).

#### Property 2

There are two kinetically distinct modes of neurotransmission [32]: one is fast and transient [33] and the other slow and sustained [34].

#### Property 3

Both modes of exocytosis can be driven by micromolar calcium concentrations, which can be achieved in the bulk cytoplasm [34]–[39].

#### Property 4

The spatial scale on which calcium entering through calcium channels triggers exocytosis is of the order of microns. The idea that microdomains of calcium control fast release of vesicles from BCs is supported by a large number of studies demonstrating that such release is effectively blocked by the slow calcium buffer ethylene glycol tetraacetic acid (EGTA) [33],[35],[36],[40],[41].

#### Property 5

For simplicity we assumed that the number of ribbons, and therefore the number of L-type calcium channels, is proportional to terminal surface area. This assumption is tentatively supported by electron microscopy (EM) studies of goldfish “mixed” BCs [42].

This model closely described the different phases of exocytosis measured in isolated BCs stimulated by a voltage-clamp step (Figure 5A and 5B) [35].

To assess the utility of the model, we compared its predictions with the experimental measurements of synaptic function illustrated in Figures 1E–1G. Variations in the initial rate of rise of presynaptic calcium signal (Figure 5C_1,2_), the initial rate of exocytosis (Figure 5D_1,2_), and the power of the calcium signal elicited by a 1 Hz stimulus could all be accounted for by variations in terminal radius, while keeping other variables constant. For instance, the initial rate of rise of calcium in response to a step stimulus decreased as 1/r while the rate of exocytosis was found to decrease linearly with r *in vivo* (Figure 1E_2_ and 1F_2_) and the model predicted the same (Figure 5C_2_ and 5D_2_). A notable feature of the model was the prediction of a secondary rise in the release rate beginning ∼150 ms after stimulus onset. A second phase of release has also been observed experimentally by monitoring glutamate release from the BC terminal electrophysiologically in a second voltage-clamped “sniffer cell” [32].

In its simplest form, the model failed to predict the exact form of the relation between the power of the SyGCaMP2 signal and terminal radius: power varied linearly with *r in vivo* (Figure 1G_2_), but the model predicted that it would vary as 1/r (Figure 5E_2_). This discrepancy could be corrected with a 3-D diffusion model that captures local calcium differences during ongoing signaling (Figure 5E_2_, inset).

We also explored predictions of the model to alterations in physiological parameters, including the possibility of a nonlinear relation between release rate and calcium concentration, and variations in the relative threshold for L-type calcium channel activation. These changes did not qualitatively alter the predicted effects of terminal volume on the gain and kinetics of signal transmission (Figure S4). The combination of experiment and modeling presented in Figures 1–5 converges on one basic idea: the volume of the presynaptic compartment is closely linked with the gain and kinetics of synaptic transmission by determining the amplitude and time-course of the presynaptic calcium transient. This fundamental property of the synaptic compartment varies across BCs (Figure 1D) and will therefore contribute to variations in the gain of the visual signal transmitted to the inner retina, as well as the way gain varies as a function of frequency.

### Variations in Contrast Adaptation in Terminals of Different Volume

The gain of signal transmission through the retina is not constant, but varies continuously according to the recent history of the stimulus [8]. Such plasticity has been studied particularly intensively in the context of adaptation after an increase in temporal contrast, which involves depression of excitatory synaptic transmission from BCs to RGCs [18],[43],[44], likely reflecting depletion of rapidly releasable vesicles within the terminal [45]–[47]. Might the size of BC terminals also impact on the process of contrast adaptation? Larger BC terminals can contain hundreds of thousands of vesicles, and these are more mobile than in conventional synapses, acting to support the continuous mode of transmission [31],[34]. It might therefore be expected that larger terminals containing more vesicles are more resistant to depression.

**Figure 6:**
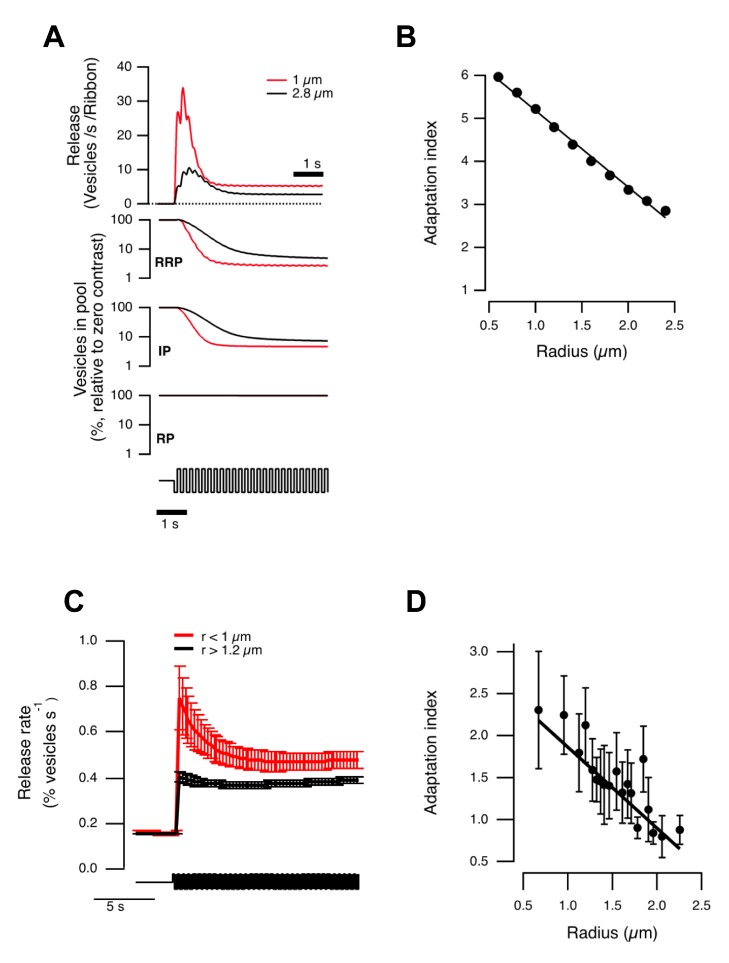
Contrast adaptation depends on terminal size: comparison of model and experiment. (A) Top, vesicle release modeled in small (red) and large (black) terminals in response to a 5 Hz stimulus (100% contrast). Small terminals are predicted to exhibit stronger adaptation. Bottom, dynamics of three vesicle pools used in the model. RRP and IP deplete faster in small terminals while RP in small and large terminals remains near constant. (B) Modeled adaptation index (Methods) decreases linearly with terminal radius. (C) Adaptation of synaptic output measured *in vivo* was more profound in smaller terminals. Graph shows release dynamics of OFF terminals with r<1 µm (red) and r>1.2 µm (black) in response to a 5 Hz stimulus (cf. (A)). (D) Adaptation index decreases linearly with terminal radius, as predicted by the model (*n* = 236 OFF terminals from seven fish, each bin is an average of 12 individual terminals). Spearman correlation = −0.86, critical value (*p* = 0.05) = 0.45. See also Figure S5.

To quantify time-dependent changes in synaptic gain we used an “Adaptation Index” (AI), calculated as the ratio between the peak initial response to an increase in contrast and the later steady-state response. Using a stimulus of 100% contrast modulated at 5 Hz, and assuming a constant density of vesicles in terminals of different volume, the model predicted that the rate of vesicle release would depress more profoundly in smaller terminals (Figure 6A and 6B). This can be understood in terms the RRP and IP depleting faster and to a lower steady-state in small terminals compared to large (Figure 6A).

Assaying vesicle release *in vivo* using sypHy confirmed that smaller terminals displayed greater depression (Figure 6C), and AI decreased linearly with r (Figure 6D). The absolute values of AI measured using sypHy were, however, lower than those predicted by the model: in the smallest terminals, an AI of ∼2.5 was measured using sypHy, while the model predicted values of ∼6. This difference is likely to reflect the relatively low time-resolution of sypHy measurements, causing us to underestimate the initial peak release rate (cf. Figure 6A). Nonetheless, the model and experimental measurements together demonstrate that the geometry of the presynaptic compartment is one of the factors determining the kinetics of vesicle depletion and, therefore, adaptation.

It has recently been demonstrated that there are two opposing forms of plasticity when the retina responds to an increase in temporal contrast: while some BCs and ganglion cells adapt, others become sensitized [18],[48]. The balance between adaptation and sensitization was also found to vary as a function of terminal size [14],[18]; on average, larger terminals tended to show less adaptation (Figure 6C) with the largest terminals exhibiting sensitization (Figure 6D). Sensitization of the BC output has been shown to result from reduced inhibition from amacrine cells [18],[19],[49], and so is not predicted by the simple one compartment model.

### Linear- and Non-linear Transformations of the Visual Signal: Impact of Terminal Volume

An empirical description of the relation between variations in the intensity of light falling on the retina and the spike-rate of ganglion cells can often be obtained using a model comprised of two stages: a linear transformation of the input feeding into a rectifying non-linearity [7],[50],[51]. How does this linear-nonlinear (LN) model map onto the retinal circuit [5]? Transformations of the visual input are roughly linear through the processes of phototransduction and transmission by the synapses of photoreceptors, as far down the visual pathway as the cell body of BCs [22],[43],[52],[53]. But what of the next neural compartment in the visual pathway—the synaptic terminal of BCs?

**Figure 7:**
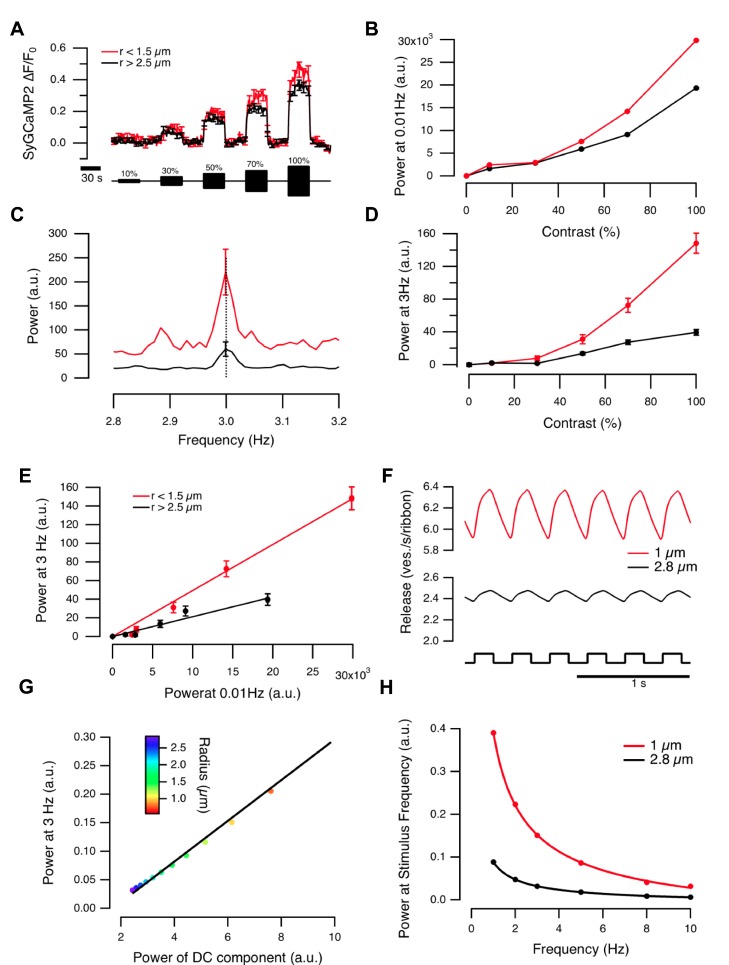
Linear and rectifying components of contrast response vary with terminal size. (A) SyGCaMP2 responses of BC terminals to stimuli of varying contrast (3 Hz). Average ΔF/F of all contrast responding terminals (both ON and OFF) with r<1.5 µm (*n* = 66 terminals, red) and r>2.5 µm (*n* = 119 terminals, black). Note how these synaptic calcium responses are strongly rectifying. (B) Power of the DC component measured at 0.01 Hz for stimuli shown in (A). The DC component was larger in small terminals (cf. Figure 4C). (C) Power spectrum of the response to 100% contrast (3 Hz) for small (red) and large (black) terminals. (D) Power at 3 Hz varies with contrast. See also Figure S4. (E) Power of the linear and DC components are directly proportional, but the proportionality coefficient is larger for smaller terminals. The Pearson correlation coefficient was 0.99 for small terminals and 0.97 for large. (F) Modeled release in response to a 3 Hz stimulus (100% contrast). (G) The model predicts that the power of the calcium response at 3 Hz is directly proportional to the power of the DC component, as was observed experimentally in (E). (H) Power of the exocytic response modeled for a range of stimulus frequencies. Power at the stimulus frequency varies as 1/f for both small and large terminals.

Measurements of vesicle release with sypHy demonstrated strong rectification in the synaptic output because an increase in stimulus variance caused an increase in the mean rate of vesicle release even when the mean luminance was held constant, as shown in Figure 6C. This behavior is predicted by the model (Figure 6A), where it reflects the rectifying relation between membrane potential and the amplitude of the calcium current (Figure 4). To test this explanation by experiment, we imaged the presynaptic calcium signal using SyGCaMP2, while applying a square wave stimulus modulated at 3 Hz. Figure 7A compares the SyGCaMP2 signal averaged over two populations of terminals that generated a significant response to this stimulus: 66 with r<1.5 µm and 119 with r>2.5 µm. In both populations the presynaptic calcium signal was a strongly rectifying function of light intensity, causing a steady increase in calcium that was graded with contrast (Figure 7B). These measurements identify voltage-dependent calcium channels in the synaptic terminal of BCs as being responsible for the first major non-linear transformation of the visual signal as it is transmitted through the retina.

Although the most obvious aspect of the SyGCaMP2 signal elicited by an increase in the variance of the stimulus was a maintained increase in presynaptic calcium, smaller fluctuations could also be detected (Figure 1G). The power of the fluctuations following a periodic stimulus was used to quantify the linear component of the synaptic calcium response [22], and the power of this linear component was ∼3-fold larger in the smaller population of terminals (Figures 1G, 7C, and 7D). Further, when the contrast of the stimulus was varied between 10% and 100%, the power of the linear component was directly proportional to the power of the DC component (Figure 7E), as predicted by the model (Figure 7G). The proportionality constant for the small population of terminals was 2.4 times that of the large population (Figure 7E), which was also predicted by the model. The relatively slow response time of SyGCaMP2 (τ_decay_∼200 ms [13]) prevented the imaging of synaptic responses following stimuli at frequencies greater than 3 Hz, but the results in Figure 7 demonstrate that smaller terminals generate relatively stronger linear responses than large terminals, and therefore encode fluctuating stimuli more effectively.

### Variations in Temporal Filtering in Terminals of Different Volume

How do variations in filtering of the presynaptic calcium signal impact on the output from the synapse? Assaying vesicle release with SypHy demonstrated that smaller terminals respond to a step of light with higher relative release rates (Figure 1F), but the resolution of this reporter was too low to detect modulations at stimulus frequencies >1 Hz (i.e., the linear component of the output). Nonetheless, the model predicted that the modulation in release rate would be directly proportional to the steady rate once the terminal had adapted (Figure 7F), and sypHy can be used to assess variations in the steady-rate of release [14]. We therefore used steady-state measurements to compare temporal filtering in the output of small and large terminals.

**Figure 8:**
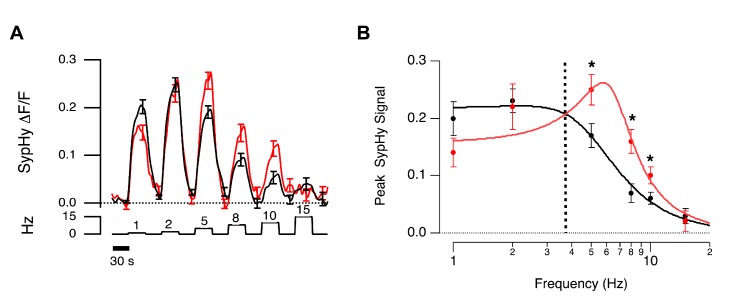
Frequency tuning of BCs with different terminal size. (A) Mean sypHy fluorescence signals in OFF terminals in response to full-field stimuli of different temporal frequencies measured *in vivo* (red, r_small_<1 µm, *n* = 10; black, r_large_>1.5 µm, *n* = 32). (B) Synaptic gain of large (black) and small (red) terminals as a function of stimulus frequency. Results are described by the function
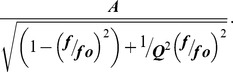
For large terminals, A = 0.22±0.02, fo = 4.96±0.45, Q = 0.78±0.17. For small terminals A = 0.16±0.02, fo = 6.43±0.27, Q = 1.57±0.37. Asterisks mark significantly different responses at a given frequency, as evaluated by Mann-Whitney U test (*p*<0.05).

Figure 8A shows averaged sypHy signals to stimuli of varying frequency, for two populations of terminals: small (r<1 µm) and large (r>1.5 µm) The amplitude of the response, reflecting the steady rate of vesicle release, is plotted as a function of frequency in Figure 8B. Small terminals transmitted frequencies between 5 and 10 Hz significantly better than large terminals. For instance, at a frequency of 8 Hz, the relative response of small terminals was ∼2.4-fold that of large.

If the power of the modulated response is proportional to the mean release rate, as suggested by the results in Figure 7E, these measurements can be considered an approximation of the “transfer function” of the visual system up to the point that BCs transmit the visual signal. We therefore described the measurements using an expression commonly used to describe the output of electrical circuits with some element of resonance [54]:
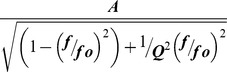
where A is a scaling factor, f_o_ is the center frequency, and Q is a quality factor describing how much the response is damped. In small terminals, Q was estimated as 1.57±0.37, while in large terminals it was 0.78±0.17. Thus small terminals more obviously maintained the bandpass characteristics of the voltage signal measured in the cell body of BCs [22], while large terminals damped out the resonant frequencies This behavior can now be understood in terms of the impact of terminal volume on the dynamics of the calcium signal controlling neurotransmitter release.

## Discussion

Many BCs transmit the visual signal through multiple terminals located in different strata of the IPL (Figure 1). Using a combination of *in vivo* imaging, slice electrophysiology, and modeling, we have found that variations in the size of these terminals will cause the signal in a single neuron to be transformed through different temporal filters as it is transmitted to the inner retina (Figures 1–4). The mechanistic link is the calcium signal that controls synaptic transmission: smaller terminals generate larger and faster calcium transients (Figures 1–5), increasing the gain of synaptic transmission and allowing higher frequencies to be transmitted (Figures 7 and 8). Diversity in the properties of synaptic transmission extend to time-dependent changes in gain: smaller terminals adapt more completely after an increase in stimulus contrast (Figure 6). These variations in synaptic properties will expand the number of processing channels that can operate in parallel through the limited number of neurons packed into a given region of the retinal network [55],[56]. These results complement recent evidence for divergence of visual channels through individual BCs, obtained by direct electrical stimulation of these neurons while monitoring the effects in multiple ganglion cells [4],[11],[57].

### Different Temporal Channels through Individual Bipolar Cells

The different temporal channels in the visual system were first distinguished by recording responses of “transient” and “sustained” ganglion cells in the retina [4]. These temporal channels partly reflect processing in the inner retina, where feedback inhibition and lateral inhibition from amacrine cells act directly on BC terminals to alter the gain and timing of the synaptic output [11],[19],[58]. Here we have described a fundamental and intrinsic property that will contribute further to the diversity of signals that BCs transmit—the geometry of the synaptic compartment [11],[55],[56].

In the future, it will be important to assess the extent to which RGCs tuned to different frequencies receive inputs from BC terminals of different sizes. Such a study will be technically demanding, requiring a detailed anatomical reconstruction of IPL circuitry. Nonetheless, Figure 1D provides the first evidence that RGCs with dendrites in different layers of the IPL will, on average, receive excitatory input from BC terminals of different sizes. For instance, layer 6 of the IPL contains the highest density of large terminals, which leads to the testable prediction that RGCs extending dendrites in this layer will be more likely to exhibit low-pass characteristics. In contrast, layer 5 has smaller terminals than the average, suggesting that RGCs collecting inputs from this layer will be tuned to higher frequencies.

### Extrinsic Factors Affecting the Frequency Response of Bipolar Cell Synapses

The purpose of the model we have presented is to provide a basic mechanistic understanding for the impact of terminal volume on calcium dynamics and vesicle release from BCs. A more comprehensive model of signal transmission from BCs would not treat the neuron in isolation but would also consider the local circuits into which the terminals are connected. In particular we have neglected the effects of direct inhibitory feedback from amacrine cells [11],[19],[58]. It is, however, notable that when the GABAergic component of this feedback was blocked, the size-dependence of release rate was maintained (Figure 2), indicating that negative feedback was not the cause of this correlation.

The temporal tuning of BCs also reflects processes in the outer retina, especially cone [59] inputs with different contact morphologies acting on dendritic glutamate receptors with different kinetics of recovery from desensitization [3],[60],[61]. The general picture that emerges is one in which amacrine cells modulate transmission of different frequency components in the visual input by acting on the background of at least two intrinsic properties varying between BCs: tuning of dendritic inputs summed at the cell body and the filter determining calcium dynamics in the synaptic terminal providing the output (Figure 3G) [62]. The key new idea that we propose here is that this synaptic filter varies between different outputs because of variations in an intrinsic property of the synaptic compartment—its volume.

### The Synaptic Compartment of Bipolar Cells as a Computational Unit

It has long been recognized that the visual system separates signals encoding different aspects of a stimulus for transmission through different pathways or “channels.” The most fundamental of these parallel representations is the separation of ON and OFF signals, which begins in distinct types of BC [63]. The separation of signals with different speeds into transient and sustained pathways also begins in BCs [6]. More than ten types of morphologically distinct BCs can be recognized in the vertebrate retina, and probably more than 20 in zebrafish [64],[65], but we still do not fully understand how these differ in their response properties. The general thinking has been that one type of BC transmits one type of signal, but more recent work indicates that it might be more fruitful to focus on the synaptic terminal as the fundamental unit of signal transmission, which would allow for divergence of different signals from the one neuron [11].

Implicit in this idea is the notion that the different synaptic outputs of a neuron are functionally isolated from each other, at least to some extent. Does the geometry of the terminal arborization allow this? Simultaneous measurements of calcium signals in connected terminals demonstrate that the answer is yes: connecting processes provide a sufficient diffusional barrier to allow calcium signals with different amplitudes and kinetics to remain local to individual terminals (Figure 3). Such isolation of presynaptic calcium signals would also allow for independent modulation of different synaptic compartments by amacrine cells. The potential number and diversity of these synaptic transformations becomes even greater if one considers that individual amacrine cells provide negative feedback though many neurites with distinct biophysical and synaptic properties [66]. Such presynaptic heterogeneity has previously been observed in sensory pathways of insects, including cricket auditory afferents [67] and *Drosophila* olfactory receptor neurons [68].

## Materials and Methods

### *In Vivo* Imaging of Presynaptic Ca^2+^ Signals and Vesicle Fusion

All procedures were carried out according to the UK Animals (Scientific Procedures) Act 1986 and approved by the UK Home Office. Transgenic zebrafish expressing SyGCaMP2.0 and sypHy were maintained on a 14/10 h light/dark cycle at 28°C. From 24 hpf the larvae were maintained in fish medium (E2) containing 1-phenyl-2-thiourea at a final concentration of 200 µM (Sigma) to prevent pigment formation. Fish were imaged as described previously [13]. Briefly, before experiments 8–10 dpf larvae were anesthetized in 0.016% Tricaine (Sigma) and immobilized in low melting point agarose. To prevent eye movement α-bungarotoxin (2 mg/ml) was injected into the extraocular space. Imaging was performed using a custom-built multiphoton microscope, equipped with a mode-locked Chameleon titanium-sapphire laser (Coherent) tuned to 920 nm and controlled using ScanImage v.3.6 software [69].

The retina was imaged through an Olympus LUMPlanFI 40× water immersion (0.8 NA) objective. Green emission from sypHy and SyGCaMP2 was collected both through the objective and through an oil condenser (ND 1.4, Olympus), filtered through GFP filters (530/50 nm, Chroma Technology), and detected with GaAsP photomultipliers (Hamamatsu). Images (128×128 pixels) were acquired every 0.128 seconds, resulting in a sampling frequency of 7.8 Hz. Full-field light stimuli were delivered using an amber LED (590 nm) filtered through a 600/10 BP filter and projected through a light guide onto the surface of the bath, close to the eye of the fish. The mean intensity of light stimuli was ∼2×10^5^ photons/µm^2^/s, which is in the low photopic range.

Importantly, optical measurements in the live retina necessarily give rise to a background activation of photoreceptors. This is due to direct laser activation of photopigment, but usually more importantly, due to indirect activation of photopigments from the emission light of excited fluophores [70]. As a result, rods are typically saturated, and cones partially adapted, giving rise to a low-photopic background light-level. Indeed, we observed similar effects in our *in vivo* experiments (Figure S1F). However, this background illumination was weaker than typically observed in e.g., rabbit [70], presumably as the zebrafish retina is cone dominated. Nevertheless, following activation of the laser scanning, we typically waited 30 seconds before commencing with additional visual stimulation to ensure the retina adapted to the background levels attributed to the laser.

### Image Analysis

Movies were processed using the SARFIA suite of analysis routines [17] running in Igor Pro 6 (Wavemetrics). We detected terminals on the basis of single images obtained by averaging many frames of the movie corresponding to total integration times of several tens of seconds. ROIs corresponding to terminals within these averaged images were defined using a filtering algorithm based on a Laplace operator followed by application of a threshold, as described in detail in [17]. This algorithm will define most or all of the ROIs that an experienced observer would recognize by eye. To prevent bias between ON and OFF terminals within a single field of view, the average image was obtained from movies in which light steps were applied, and/or light intensity modulated at 1 Hz or faster.

### Estimation of Terminals Size by Two-Photon Imaging

The point-spread function of the used microscope in XY dimension was 0.5 µm and terminals appeared larger when we increased intensity of the 2-photon laser. We therefore tested whether terminals might appear smaller when less active, but found that this was not the case (as described in Figure S1 and Text S1). Average terminal sizes were similar between different fish (Figure S5A) and the effects of terminal size on adaptation kinetics could be observed in individual fish (Figure S5B) as well as in data averaged over multiple fish (Figure 6C). However, the distribution of estimated terminal sizes in SyGCaMP2 was shifted to larger values, compared to sypHy or Synaptophysin-EGFP fish (Figure S1H). This is likely explained by the greater overall brightness of SyGCaMP2. The z-resolution of the microscope was ∼2 µm. Given an average radius of just above 1 micron per terminal, this resolution was therefore large enough to avoid underestimating the size of terminals traversed non-centrally by the optical plane, but small enough to avoid out-of-focus terminals contributing to the signal.

### Statistical Analysis

All statistical analysis was performed in Igor Pro 7 (Wavemetrics). Differences in different parameters in large and small terminals, described in Figure 3, were analyzed using Wilcoxon signed-rank test. This test was chosen because some responses of large and small terminals were recorded from the same neuron. Sample sizes were not determined a priori. Analysis of the response dynamic was automatic and no knowledge of terminal size was used until the last moment. All animals demonstrating robust response to light were included in the analysis. Figure 1B represents biological replicate, representative from more than five fields of view. No lack of reproducibility was found. All error bars in figures show ± 1 SEM, unless stated otherwise in the legend.

### Calculation of Vesicle Release Rates

V′_exo_, the fraction of total vesicles in the terminal released per second, was calculated from the sypHy signal according to the equation:
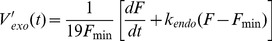
(1)where F is the average fluorescence intensity over the terminal at time t, F_min_ is the intensity when the rate of vesicle release is at a minimum, and k_endo_ is the rate-constant of vesicle retrieval. The calculation of this formula is described in [14],[18]. Estimation of V′_exo_ requires differentiation of the sypHy trace, which in turn amplifies noise, so Equation 1 was applied after smoothing with a series of single or double exponential functions to obtain “non-noisy” traces before calculation of V′_exo_. These fits are shown in Figures 1F and 5C.

### Slice Electrophysiology and Imaging

Slices of goldfish retina were perfused with extracellular solution containing (in mM) 120 NaCl, 2.5 CaCl_2_, 2 KCl, 1 MgCl_2_, 0.1 CaCl_2_, 4 HEPES, 10 glucose (pH 7.7, 255 mOsm). Experiments were carried out at room temperature and slices visualized under oblique infrared illumination through a 60× objective (NA 0.9) on an upright microscope. Whole cell recordings were obtained from “large” terminals (diameter: 4–12 µm) or from the soma of “mixed” BCs using 8–12 MΩ patch electrodes [71]. For this, we targeted large terminals in layers 5/6. The intracellular solution contained (in mM) 104 Kgluconate, 8 KCl, 2 MgCl_2_, 4 HEPES, 0.5 EGTA, 2 MgATP, 1 NaGTP, 1 NacGMP (pH 7.4, 250 Osm), and 100 µM of the hexapotassium salt of the low affinity Ca^2+^ indicator OGB-5N. Recordings were left for 1–5 min to allow time for the Ca^2+^-indicator to diffuse into adjacent small terminals (diameter 1–4 µm) see also [20]. Series resistance ranged from 8–15 MΩ while input resistance was >1 GΩ at −70 mV. Recordings were corrected for junction potentials (calculated as −11.95 mV).

OGB-5N was imaged at 40 Hz using an electron multiplying charge coupled device (EM CCD) camera (Hamamatsu C9100). Subsequent image analysis was performed using ImageJ, Igor Pro, and Matlab. OGB-5N signals were quantified as changes in fluorescence relative to background fluorescence at each pixel (ΔF/F_o_) and converted to estimates of absolute Ca^2+^ concentration:
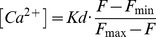
(2)F_max_/F_min_ of OGB-5N is ∼14.7. Since resting Ca^2+^ in bipolar terminals (∼50 nM [30]) is much lower than the K_d_ of OGB-5N (20 µM) we assumed that F_rest_ = F_min_.

## Supporting Information

Figure S1 **Size estimation does not depend on the stimulus condition (related to** **Figure 1** **).** (A) Two images of the same field of view: the left obtained from an average of 45 s in the dark, and the right averaged over a 45 s period in which the mean light intensity was in the low photopic range, including 45 s of temporal contrast applied at 1 Hz (100% contrast). Scale bar 10 m. (B) OFF terminals have brightest fluorescence during contrast presentation and dimmest fluorescence when exposed to steady light. Black bars show parts of the movie used for image averaging. (C) Distribution of terminal sizes estimated from averages shown in (A) (at steady light, red and temporal contrast, black). (D) Cumulative distributions, calculated from (C). (E) Terminal sizes estimated from terminals during steady and flickering light stimulation (c.f. (A)). Each point represents size estimation of an individual terminal. All points are scatted around a line through the origin with slope of 1, suggesting that size estimation is not affected significantly by the terminal activity. (F) Example of *n* = 589 OFF cells responding to laser and visual stimulation (arrows). Top: average of all responses, bottom: individual responses. Error in (standard error of the mean) SEM. (G) Responses of OFF terminals of different sizes to light decrement. Bins are the same as in Figure 1E. (I) Distributions of terminal radii calculatEed from individual layers.
(TIF)

Figure S2 **Recording “mixed” BCs from the soma (related to** **Figure 2** **).** To ensure that the size dependence of current evoked calcium signals measured in different terminals belonging to the same “mixed” BC was not dependent on the position of the micropipette, we repeated experiments shown in Figure 2D–2F but this time targeted the soma of individual cells rather than the large terminal. The size dependence persisted in somatal recordings. One example of *n* = 4 is shown (c.f. Figure 2D–2F).
(TIF)

Figure S3 **A 3-D diffusion model of Ca^2+^ in the bipolar cell synapse (related to** **Figure 3** **).** (A–C) Predicted calcium levels at different distances from a hotspot in a 5 µm (black) and a 1 µm (red) radius spherical compartment, shown at three different time-scales. (D) Estimated release rates driven by calcium as shown in (C). (E) Concentration of unbound “fixed” (grey) and “diffusible” (green) buffers under normal buffering conditions during step depolarisation of a 1 micron radius terminal (c.f. red in (B)). The dotted line indicates unbound buffer concentration at the channel mouth (“hotspot”), which the solid line indicates concentration at the center of the compartment. The coloration indicates the possible range of unbound buffer concentration at different locations within the compartment. (F) Corresponding calcium concentration at the hotspot (dotted) and globally (solid). (G, H) as (E, F) but with 10 times elevated “fixed” buffer concentration. Elevating the fixed buffer has only small effects on the kinetics of calcium free calcium concentration, but does affect peak calcium concentration at the hotspot.
(TIF)

Figure S4 **Modulation of release with changed ICa threshold and calcium dependence of release: predictions of the model (related to** **Figure 4** **).** (A, B, left) Modeled calcium (A) and release (B) in response to a 3 Hz flickering stimulus from an r = 1 micron compartment with different thresholds for activation of the L-type calcium current (V_rest_ always = −44 mV). The threshold was increased (light green) and decreased (dark green) from the value used in the main model (red) by 3 mV in each case. Right: modulation amplitude of calcium (A) and release (B) quantified for the three threshold conditions in different size compartments. Changing the threshold had only minimal effect on the overall size dependence of calcium and release modulation. (**C, D**) Steady state modulation of modeled calcium (C) and release (D) in an r = 1 micron compartment in response to an ongoing 3 Hz stimulus. Changing the Hill coefficient for calcium dependence of release from 1 (linear = in main model, red) to 3 (cooperative, light blue) systematically increases the modulation amplitude of release (D, left) across all frequencies tested (D, right).
(TIF)

Figure S5 **Comparison of terminal sizes and adaptation dynamics in different fish (related to** **Figure 5** **).** (A) Average terminal sizes were similar in each of six different fish. (B) Adaptation to temporal contrast (100%, 5 Hz) in a single fish. Smaller terminals (red) respond with higher gain and adapt more profoundly than large (black), in a manner similar to the behavior averaged over 6 fish (Figure 5C). (C) Same as Figure 5C, but on a longer time scale. Contrast facilitation is more pronounced in larger terminals.
(TIF)

Figure S6 **Active voltage spikes should boost high frequency components (related to** **Figure 6** **).** (A) A “chirp” stimulus modulating at 100% contrast ramping from 0.1 to 20 Hz and back down again over a period of 10 s (top) was convolved with the same impulse response used in Figure 3 to yield a prediction of the generator potential. (B) Addition of Brownian motion noise (standard deviation [SD] = 1.4 mV) was used to yield an estimate of membrane voltage. Two separate predictions were drawn from the model at this point: graded (black) and spiking (red). A threshold was added to the “membrane voltage” trace to predict spikes, which occurred with an exponential refractory period of 300 ms. Spike amplitude was fixed at 20 mV, with a half width of 3 ms. (C) Bulk calcium and (D) release was calculated as before (Figure 4) from the graded and spiking voltage traces. (E) Average release rates of 100 graded (black) and 100 spiking (red) model BCs. Note that the mean frequency response of the spiking system is highly reminiscent of the generator frequency response (A), while the graded system imposes a powerful low pass filter on the signal.
(TIF)

Table S1 **List of parameters used in single compartment model.**
(DOCX)

Table S2 **List of parameters used in 3-D model.**
(DOCX)

Data S1 **All raw data files as well as averages and statistical parameters presented in the manuscript.** Formats provided include raw-text (Ascii) and Excel (xls). In addition, we provide the original Igor-Pro files (Wavemetrics), which contain both the raw data and the original figure formatting. Data referring to particular panels are located in the respective folders. For any further information, please contact Tom Baden at thomas.baden@uni-tuebingen.de.
(ZIP)

Text S1 **Supplemental information.** (1) Variations in estimates of terminal size were not correlated with variations in terminal brightness. (2) Modeling presynaptic calcium dynamics and vesicle release. (3) A 3-D model predicting calcium spread at the channel mouth. (4) The effect of changing L-type calcium channel threshold and calcium dependency of release: predictions of the model. (5) Encoding of high frequency components using spikes. (6) Supplemental references.
(DOCX)

## Abbreviations

AI: adaptation index
BC: bipolar cell
DC: direct current
dpf: days post fertilization
EGFP: enhanced green fluorescent protein
IP: intermediate pool
IPL: inner plexiform layer
OGB: Oregon green BAPTA
RGC: retinal ganglion cell
ROI: region of interest
RP: reserve pool
RRP: readily releasable pool
