# Supplementary figures and images for: A Synaptic Mechanism for Temporal Filtering of Visual Signals
Source: PLoS Biol. 2014 Oct 21;12(10):e1001972. doi: 10.1371/journal.pbio.1001972 (PMC4205119; doi:10.1371/journal.pbio.1001972)

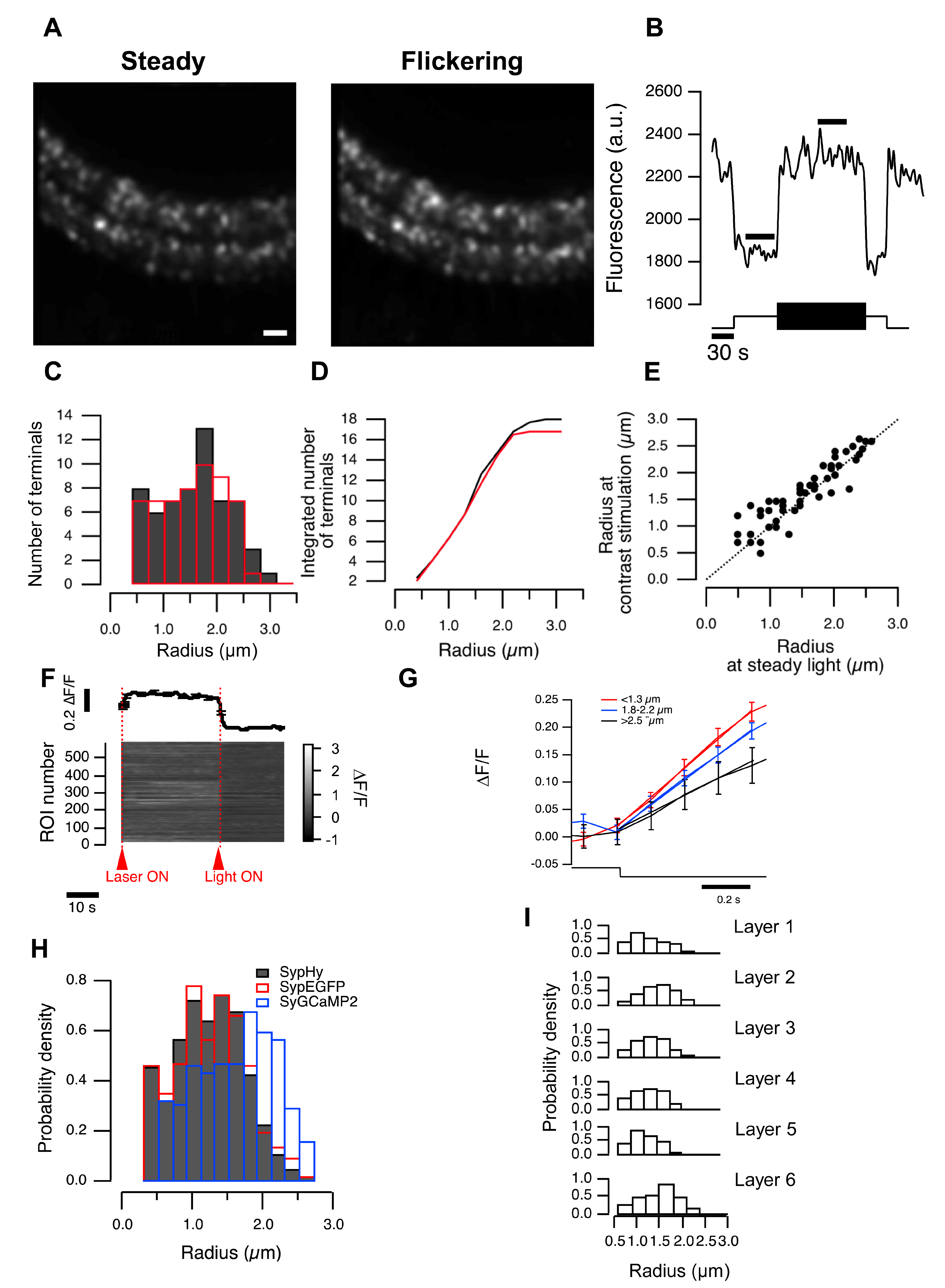

Supplement: Figure S1 — Size estimation does not depend on the stimulus condition (related to Figure 1 ). (A) Two images of the same field of view: the left obtained from an average of 45 s in the dark, and the right averaged over a 45 s period in which the mean light intensity was in the low photopic range, including 45 s of temporal contrast applied at 1 Hz (100% contrast). Scale bar 10 m. (B) OFF terminals have brightest fluorescence during contrast presentation and dimmest fluorescence when exposed to steady light. Black bars show parts of the movie used for image averaging. (C) Distribution of terminal sizes estimated from averages shown in (A) (at steady light, red and temporal contrast, black). (D) Cumulative distributions, calculated from (C). (E) Terminal sizes estimated from terminals during steady and flickering light stimulation (c.f. (A)). Each point represents size estimation of an individual terminal. All points are scatted around a line through the origin with slope of 1, suggesting that size estimation is not affected significantly by the terminal activity. (F) Example of n = 589 OFF cells responding to laser and visual stimulation (arrows). Top: average of all responses, bottom: individual responses. Error in (standard error of the mean) SEM. (G) Responses of OFF terminals of different sizes to light decrement. Bins are the same as in Figure 1E. (I) Distributions of terminal radii calculatEed from individual layers. (TIF) [file pbio.1001972.s001.tif]

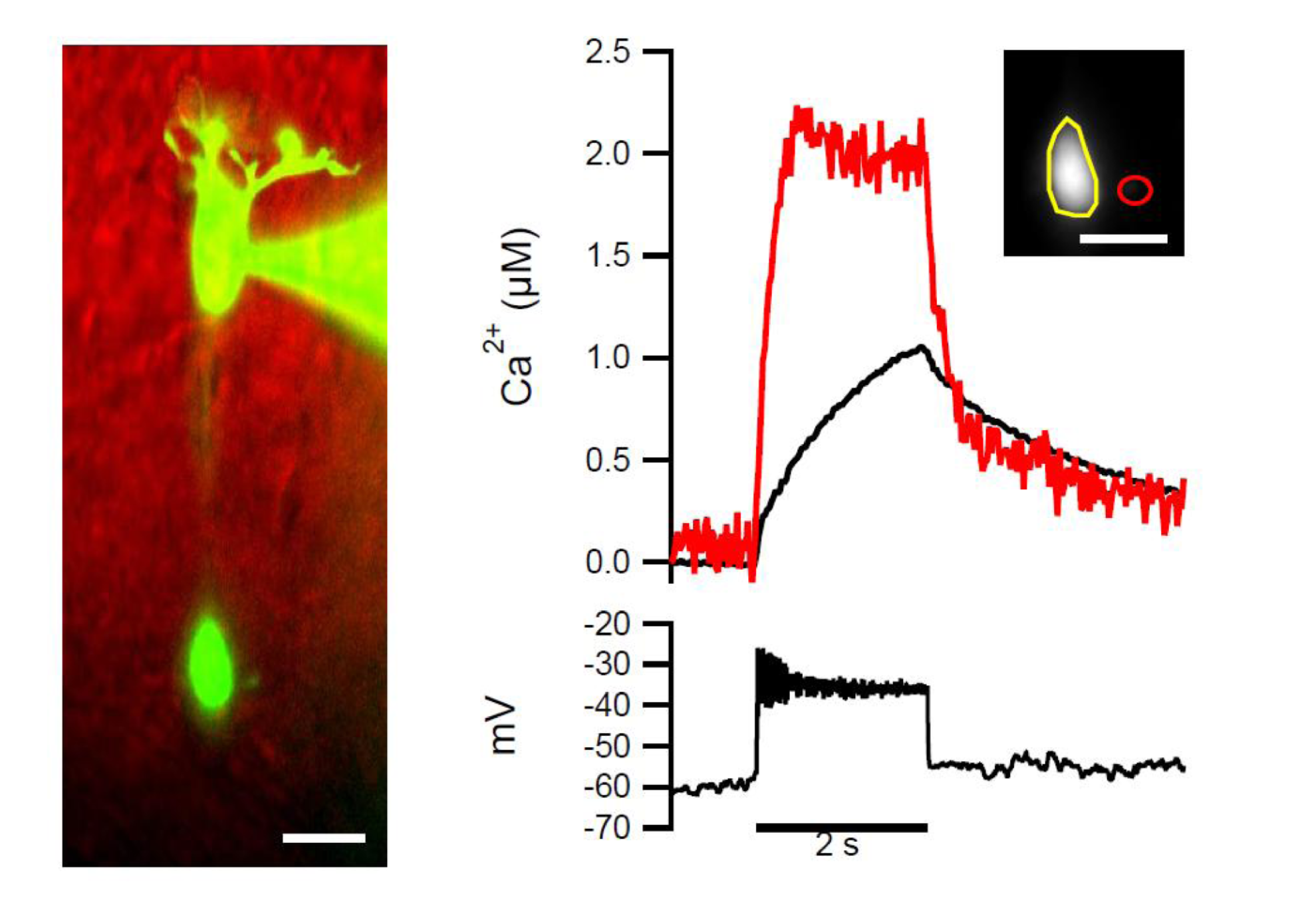

Supplement: Figure S2 — Recording “mixed” BCs from the soma (related to Figure 2 ). To ensure that the size dependence of current evoked calcium signals measured in different terminals belonging to the same “mixed” BC was not dependent on the position of the micropipette, we repeated experiments shown in Figure 2D–2F but this time targeted the soma of individual cells rather than the large terminal. The size dependence persisted in somatal recordings. One example of n = 4 is shown (c.f. Figure 2D–2F). (TIF) [file pbio.1001972.s002.tif]

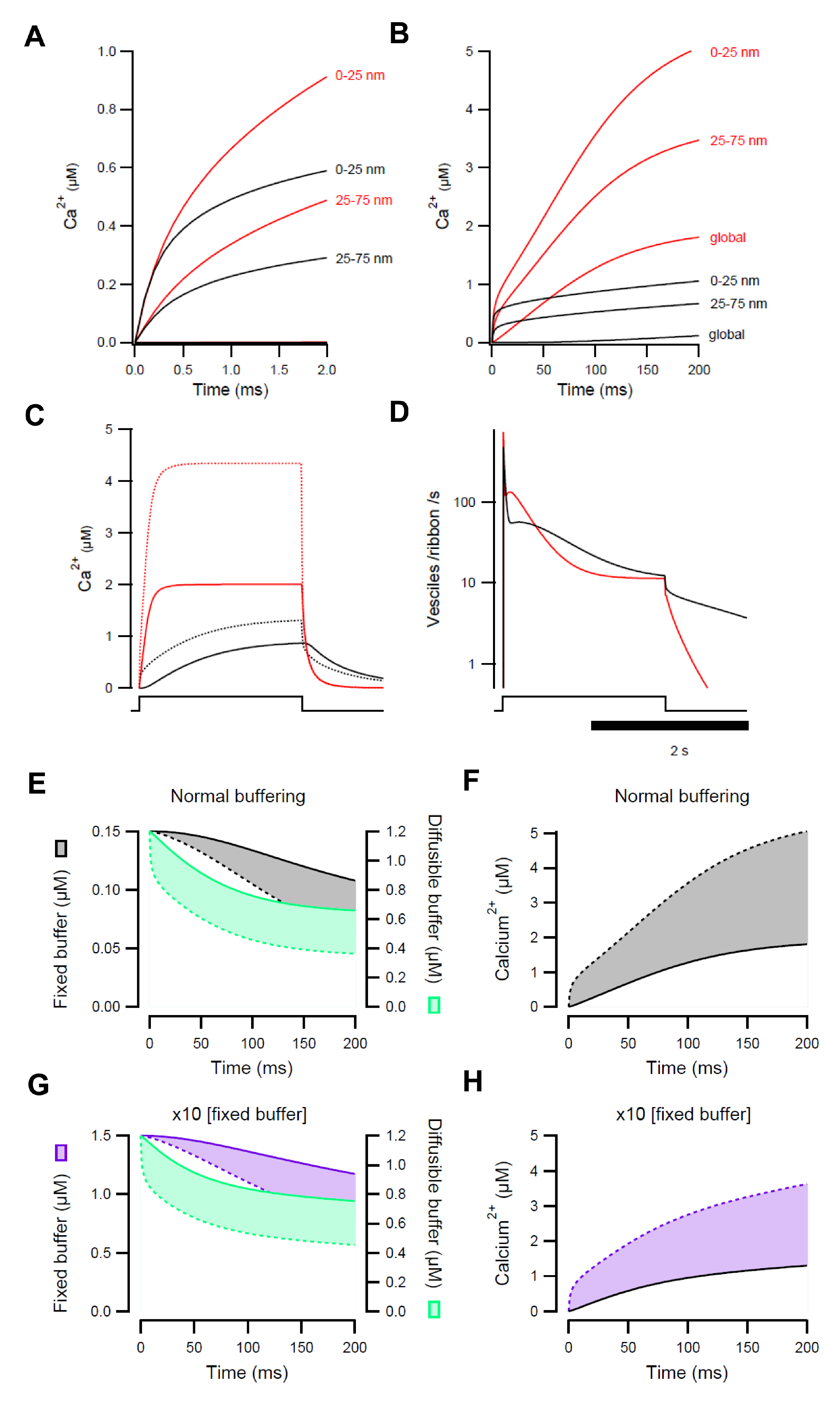

Supplement: Figure S3 — A 3-D diffusion model of Ca2+ in the bipolar cell synapse (related to Figure 3 ). (A–C) Predicted calcium levels at different distances from a hotspot in a 5 µm (black) and a 1 µm (red) radius spherical compartment, shown at three different time-scales. (D) Estimated release rates driven by calcium as shown in (C). (E) Concentration of unbound “fixed” (grey) and “diffusible” (green) buffers under normal buffering conditions during step depolarisation of a 1 micron radius terminal (c.f. red in (B)). The dotted line indicates unbound buffer concentration at the channel mouth (“hotspot”), which the solid line indicates concentration at the center of the compartment. The coloration indicates the possible range of unbound buffer concentration at different locations within the compartment. (F) Corresponding calcium concentration at the hotspot (dotted) and globally (solid). (G, H) as (E, F) but with 10 times elevated “fixed” buffer concentration. Elevating the fixed buffer has only small effects on the kinetics of calcium free calcium concentration, but does affect peak calcium concentration at the hotspot. (TIF) [file pbio.1001972.s003.tif]

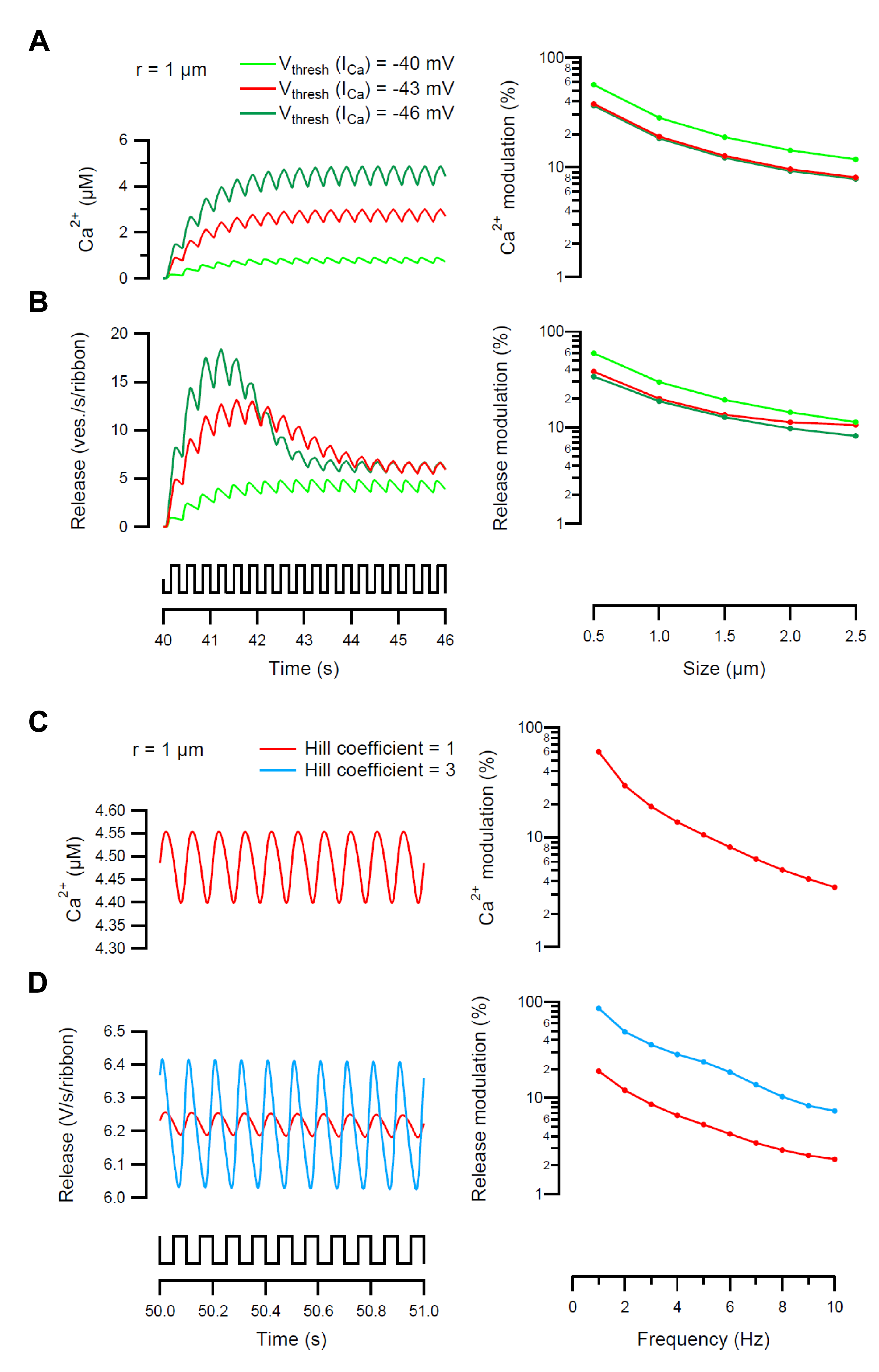

Supplement: Figure S4 — Modulation of release with changed ICa threshold and calcium dependence of release: predictions of the model (related to Figure 4 ). (A, B, left) Modeled calcium (A) and release (B) in response to a 3 Hz flickering stimulus from an r = 1 micron compartment with different thresholds for activation of the L-type calcium current (Vrest always = −44 mV). The threshold was increased (light green) and decreased (dark green) from the value used in the main model (red) by 3 mV in each case. Right: modulation amplitude of calcium (A) and release (B) quantified for the three threshold conditions in different size compartments. Changing the threshold had only minimal effect on the overall size dependence of calcium and release modulation. (C, D) Steady state modulation of modeled calcium (C) and release (D) in an r = 1 micron compartment in response to an ongoing 3 Hz stimulus. Changing the Hill coefficient for calcium dependence of release from 1 (linear = in main model, red) to 3 (cooperative, light blue) systematically increases the modulation amplitude of release (D, left) across all frequencies tested (D, right). (TIF) [file pbio.1001972.s004.tif]

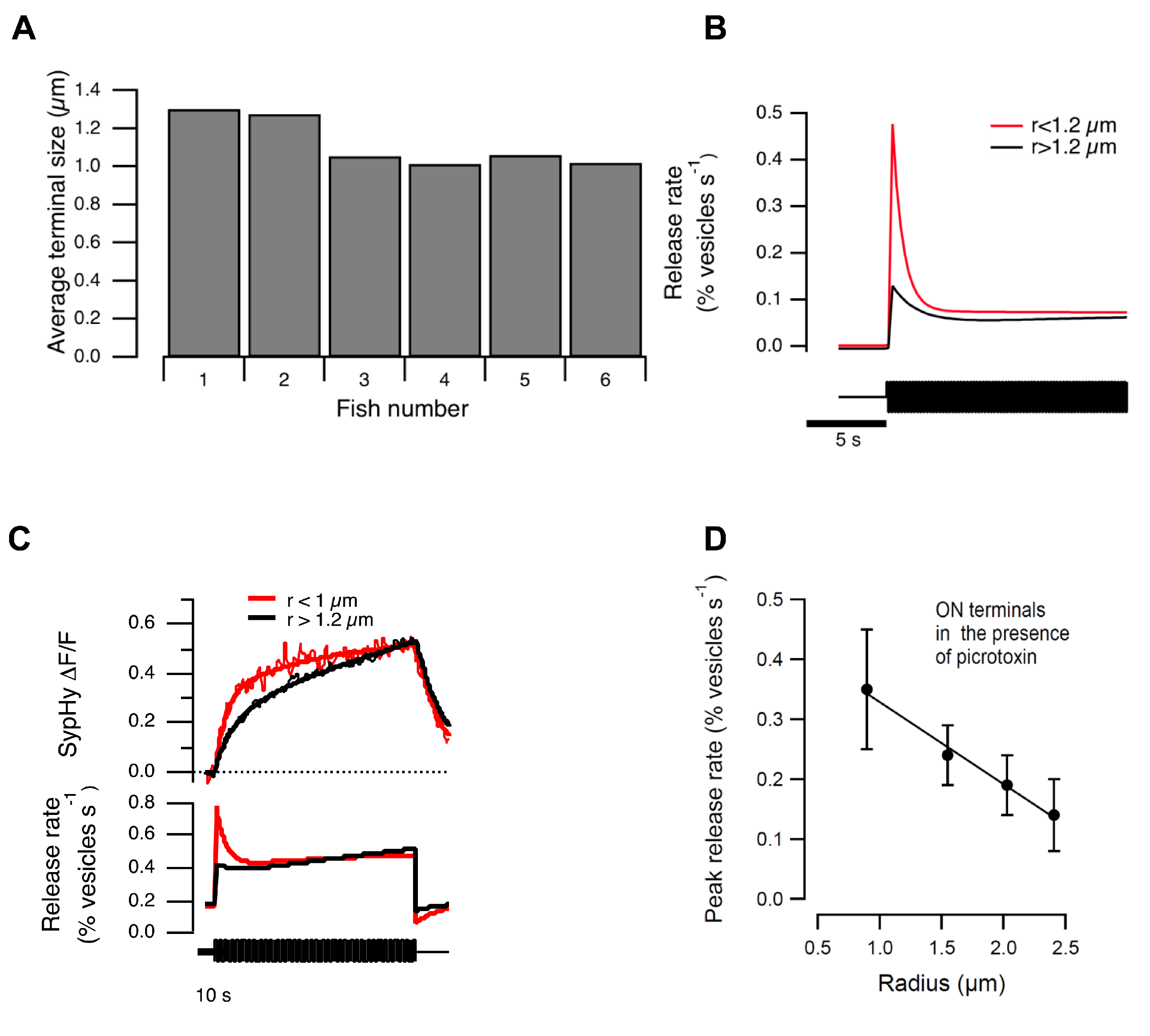

Supplement: Figure S5 — Comparison of terminal sizes and adaptation dynamics in different fish (related to Figure 5 ). (A) Average terminal sizes were similar in each of six different fish. (B) Adaptation to temporal contrast (100%, 5 Hz) in a single fish. Smaller terminals (red) respond with higher gain and adapt more profoundly than large (black), in a manner similar to the behavior averaged over 6 fish (Figure 5C). (C) Same as Figure 5C, but on a longer time scale. Contrast facilitation is more pronounced in larger terminals. (TIF) [file pbio.1001972.s005.tif]

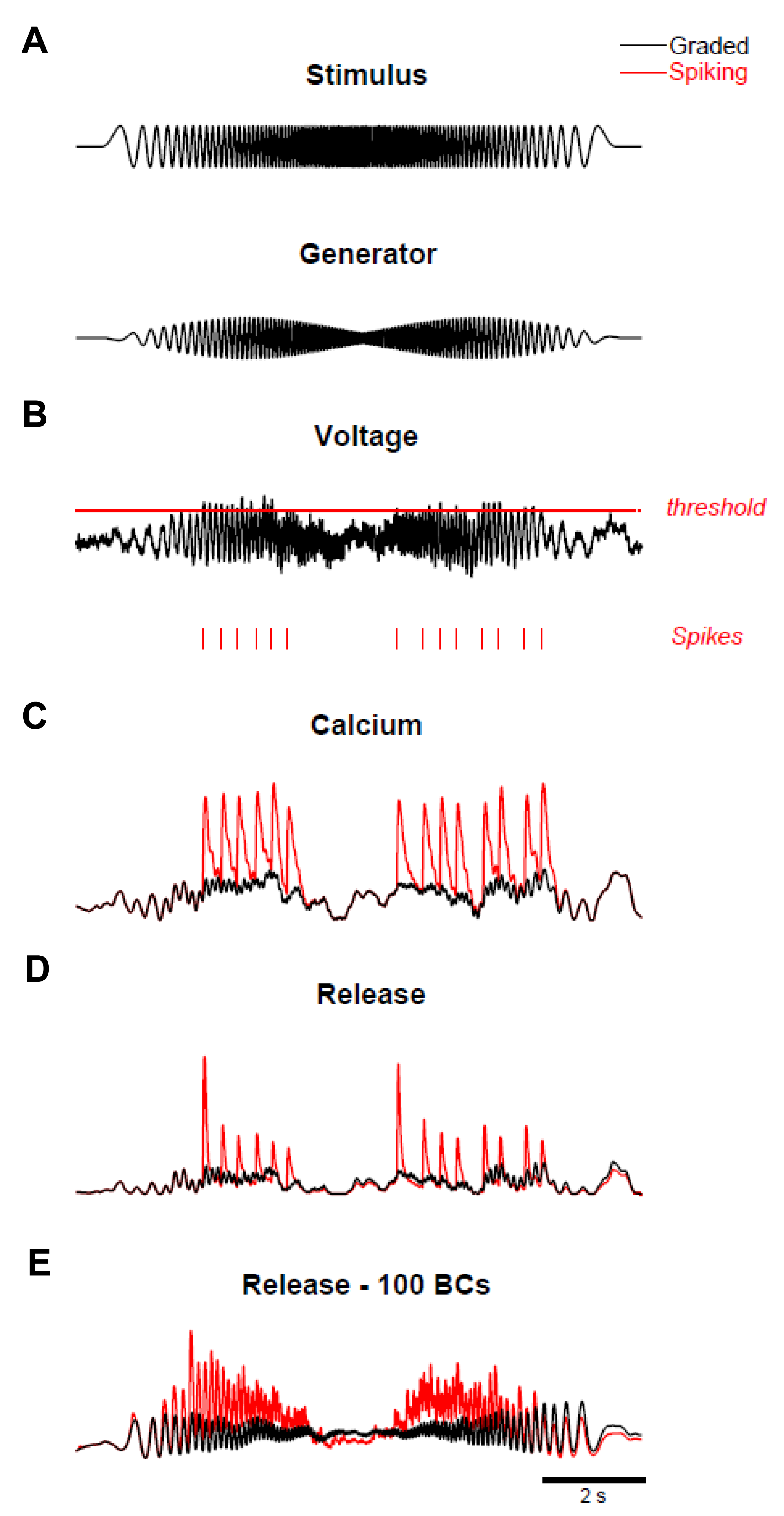

Supplement: Figure S6 — Active voltage spikes should boost high frequency components (related to Figure 6 ). (A) A “chirp” stimulus modulating at 100% contrast ramping from 0.1 to 20 Hz and back down again over a period of 10 s (top) was convolved with the same impulse response used in Figure 3 to yield a prediction of the generator potential. (B) Addition of Brownian motion noise (standard deviation [SD] = 1.4 mV) was used to yield an estimate of membrane voltage. Two separate predictions were drawn from the model at this point: graded (black) and spiking (red). A threshold was added to the “membrane voltage” trace to predict spikes, which occurred with an exponential refractory period of 300 ms. Spike amplitude was fixed at 20 mV, with a half width of 3 ms. (C) Bulk calcium and (D) release was calculated as before (Figure 4) from the graded and spiking voltage traces. (E) Average release rates of 100 graded (black) and 100 spiking (red) model BCs. Note that the mean frequency response of the spiking system is highly reminiscent of the generator frequency response (A), while the graded system imposes a powerful low pass filter on the signal. (TIF) [file pbio.1001972.s006.tif]
