# Supplementary material for: A Synaptic Mechanism for Temporal Filtering of Visual Signals
Source: PLoS Biol. 2014 Oct 21;12(10):e1001972. doi: 10.1371/journal.pbio.1001972 (PMC4205119; doi:10.1371/journal.pbio.1001972)
Supplement: Table S1 — List of parameters used in single compartment model. (DOCX) [file pbio.1001972.s007.docx]

**Table S1. List of parameters used in single compartment model**

| τ_rise_ | 70 ms | Rise time constant of cone impulse response |
| --- | --- | --- |
| τ_decay_ | 70 ms | Decay time constant of cone impulse response |
| τ_phase_ | 100 ms | Phase delay of cone impulse response |
| \| φ \| \| --- \| | -π/5 | Phase |
| I_Ca/R_ | 3 pA | Peak calcium current at single ribbon |
| V_thresh_ | -43 mV | Threshold for calcium current activation |
| S_IV_ | -6.6 mV^-1^ | Inverse of exponent slope for calcium channel activation |
| z | 1.602 * 10^-19^ | net charge of one electron |
| A | 6.02 * 10^-23^ | Avogadro's number |
| \| κ \| \| --- \| | 800 | Calcium buffer fraction |
| τ_rise(Ca)_ | 30 ms | Rise time constant of calcium impulse response |
| τ_rad_ | 755 ms µm^-1^ | dependency of τ_decay(Ca)_ on radius (Figure 2J) |
| F_Endo_ | 0.1 s^-1^ | Fraction of vesicles endocytosed per second |
| D_V_ | 1050 µm^-3^ | Vesicle density in RP |
| RRP_max_ | 14 vesicles ribbon^-1^ | Maximal vesicles in the RRP per ribbon |
| IP_max_ | 48 vesicles ribbon^-1^ | Maximal vesicles in the IP per ribbon |
| J_RP_to_IP_max_ | 3 vesicles s^-1^ ribbon^-1^ | Maximal rate of filling of IP from RP |
| J_IP_to_RRP_max_ | 100 vesicles s^-1^ ribbon^-1^ | Maximal rate of filling of RRP from IP |
| K_IP_ | 2 μM | Ca^2+^ dependence of filling of RRP from IP |
| J_RRP_ | 3600 ves. s^-1^ rib.^-1^ μM_(Ca2+)_^-1^ | Rate of exocytosis per μM Ca^2+^ |
