# Supplementary material for: A Synaptic Mechanism for Temporal Filtering of Visual Signals
Source: PLoS Biol. 2014 Oct 21;12(10):e1001972. doi: 10.1371/journal.pbio.1001972 (PMC4205119; doi:10.1371/journal.pbio.1001972)
Supplement: Table S2 — List of parameters used in 3-D model. (DOCX) [file pbio.1001972.s008.docx]

**Table S2. List of parameters used in 3D model.**

| J_Ch_ | 3 pA per active zone: 133 pA μm-2 | Ca^2+^ influx due to channels |
| --- | --- | --- |
| J_Leak_ | 74 μM μm^-2^ s^-1^ | Ca^2+^ influx due to leak |
| P_max_ | 14817 μM μm^-2^ s^-1^ | Peak rate of Ca^2+^ extrusion |
| K_p_ | 10 μM | Dissociation constant of extrusion mechanism |
| [Ca^2+^]_rest_ | 0.05 μM | Concentration of internal Ca^2+^ at rest |
| DCa^2+^ | 220 μm s^-1^ | Diffusion coefficient of Ca^2+^ |
| [B_diff_] | 1.2 mM | Total concentration of diffusible buffer |
| K_d (diff)_ | 2.2 μM | Dissociation constant of diffusible buffer |
| K^+^_(diff)_ | 20 μM^-1^ s^-1^ | Forward rate of Ca^2+^ binding of diffusible buffer |
| D_B(diff)_ | 20 μm s^-1^ | Diffusion coefficient of diffusible buffer |
| [B_fixed_] | 0.15 mM | Total concentration of fixed buffer |
| K_d_ _(fixed)_ | 2.2 μM | Dissociation constant for Ca^2+^ of fixed buffer |
| K^+^_(fixed)_ | 2 μM^-1^ s^-1^ | Forward rate of Ca^2+^ binding of fixed buffer |
