# Supplementary material for: A Synaptic Mechanism for Temporal Filtering of Visual Signals
Source: PLoS Biol. 2014 Oct 21;12(10):e1001972. doi: 10.1371/journal.pbio.1001972 (PMC4205119; doi:10.1371/journal.pbio.1001972)
Supplement: Text S1 — Supplemental information. (1) Variations in estimates of terminal size were not correlated with variations in terminal brightness. (2) Modeling presynaptic calcium dynamics and vesicle release. (3) A 3-D model predicting calcium spread at the channel mouth. (4) The effect of changing L-type calcium channel threshold and calcium dependency of release: predictions of the model. (5) Encoding of high frequency components using spikes. (6) Supplemental references. (DOCX) [file pbio.1001972.s010.docx]

**Supplementary Materials**

**A synaptic mechanism for temporal filtering of visual signals**

Tom Baden^1^, Anton Nikolaev^1^, Federico Esposti, Elena Dreosti, Benjamin Odermatt and Leon Lagnado^2^*

MRC Laboratory of Molecular Biology, Neurobiology Division, Francis Crick Avenue, Cambridge CB2 0QH, UK

^2^School of Life Sciences, University of Sussex, Falmer, Brighton BN1 9QG, UK

^1^These authors contributed equally to this work

*To whom correspondence should be addressed at [L.Lagnado@sussex.ac.uk](mailto:L.Lagnado@sussex.ac.uk)

**Supplemental Information**

**1. Variations in estimates of terminal size were not correlated with variations in terminal brightness**

**2. Modeling presynaptic calcium dynamics and vesicle release.**

**3. A 3D model predicting calcium spread at the channel mouth.**

**4. The effect of changing L-type calcium channel threshold and calcium dependency of release: predictions of the model**

**5. Encoding of high frequency components using spikes**

**6. Supplemental references**

**1. Variations in estimates of terminal size were not correlated with variations in terminal brightness**

Might there be a bias for smaller terminals expressing SypHy or SyGCaMP2 to be detected only when the fluorescence signal is strong enough? Such an effect could contribute to the finding that smaller terminals show stronger signals from these reporters (Figure 1E-G), but two lines of evidence indicated that variations in neural activity did not cause a systematic bias in estimates of terminal size. First, the distribution of terminal sizes was very similar when measured *in vivo* in transgenic fish expressing ribeye:synaptophysin-EGFP (Figure 1C, red bars) compared to fish expressing SypHy (Figure 1C, black bars). Second, there was no significant change in estimates of size within individual terminals when their fluorescence varied between a stimulus-dependent minimum and maximum (Figure S1). Estimates of terminal sizes under different stimulus conditions are shown in Figure S1. Panel A shows the same field of view in steady light (left), and during application of temporal contrast (100%, right). Panel S1B shows the average fluorescence of OFF terminals in this field of view while these stimuli were applied: in steady light fluorescence was at a minimum, while during temporal contrast it was at a maximum. Estimates of size in steady light versus fluctuating light are plotted in Figure S1C, with each point representing an individual terminal. The results are scatted about a line through the origin with a slope of 1, leading to the conclusion that activity-dependent changes in activity did not cause any systematic bias in estimates of terminal size. Similar results were obtained in three other fish.

**2. Modeling presynaptic calcium dynamics and vesicle release**

Modulation of synaptic transmission from bipolar cells in response to light was modeled in two stages. The first was an analytical model of calcium dynamics in the presynaptic terminal. The second stage was a numerical model of Ca^2+^ driving vesicle release from ribbons, which was implemented using Igor Pro (Wavemetrics) and evaluated at a resolution of 0.1 ms time-steps.

**2.1 Membrane voltage in bipolar cells:** To account for largely linear processing upstream of bipolar cell terminals[[1](#_ENREF_1)], the light stimulus was convolved with a typical bipolar cell dendritic compartment impulse response using equation 7 in [[2](#_ENREF_2)]:

| $Cone\_impulse=\frac{-\left(\frac{t}{\tau_{rise}}\right)^{3}}{1+\left(\frac{t}{\tau_{rise}}\right)}\cdot e^{-\left(\frac{t}{\tau_{decay}}\right)^{2}}\cdot\cos\left(\frac{2\pi t}{\phi}+\tau_{phase}\right)$ | (1) |
| --- | --- |

The dynamics of the cone impulse response were specifically set to resemble our own electrophysiological measurements of responses in bipolar cells of goldfish by using τ_rise_ = 70 ms; τ_decay_ = 70 ms; τ_phase_ = 100 ms and $\varphi$ = -$\pi$/5 (Figure 5 of [[3](#_ENREF_3)]). This impulse response describes a bandpass filter with peak transmission at a little below 10 Hz. Our electrophysiological measurements in goldfish bipolar cells describe similar bandpass characteristics, with peak transmission at frequencies between 5 and 12 Hz. It should be noted that while Schnapf et al.[[2](#_ENREF_2)] measured t_rise_ = of 25-45 ms in macaque, we used a value of 70 ms to account for the slower response in goldfish.

The impulse responses of cones providing inputs to ON and OFF bipolar cells were simply inversions of each other. Convolution of the cone impulse response with the stimulus and subsequent subtraction of resting potential provided a prediction of the membrane voltage as a function of time, V_m_(t’). V_rest_ = -42 mV and -44 mV for OFF and ON terminals, respectively. This generator potential was independent of terminal size:

| $Vm(t')=Vrest+\int_{0}^{\infty} A\cdot Cone\_impulse(t)\cdot Stim(t-t')dt'$ | (2) |
| --- | --- |

where *A* is a scaling factor = 10^3^ and *Stim* is the stimulus ranging between 0 and 1. As a result, V_m_ could vary over a range of ~25 mV (-60 to -35 mV).

**2.2 Terminal geometry:** Terminals were assumed to be spherical with radii (r) varying between 0.6 - 3.0 µm. In bipolar cells, calcium influx occurs primarily through clusters of calcium channels associated with individual ribbons [[4](#_ENREF_4)] and with an estimated peak calcium current per ribbon (*I_Ca/R_*) of 3 pA [[5](#_ENREF_5)]. The number of ribbons (*nR*), and, therefore, the total peak calcium current (*peakI_Ca_*) was taken to be directly proportional to the surface area of the terminal (*SA = πr^2^*), such that terminals of 1 and 5 micron radius contained 2 and 50 ribbons [[6](#_ENREF_6)] and

| $peakI_{Ca}=nR\cdot I_{Ca}/R$ | (3) |
| --- | --- |

The volume of the terminal available to calcium ions (*V*) was calculated as

| $V=\frac{4}{3}\pi\cdot(r^{3}-(r\log r)^{3})$ | (4) |
| --- | --- |

where *r* is the radius of the terminal. The term *–(rlog r)^3^* was introduced to account for the central zone of the terminal which is not available to diffusible species because it is occupied by mitochondria[[7](#_ENREF_7)].

**2.3 Calcium current:** The generator potential controlled the opening of L-type calcium channels in the synaptic terminal based on the measured I-V relation and the peak I_Ca_[[8](#_ENREF_8)], so that the calcium current as a function of time could be calculated as :

| $I_{Ca}(t)=peakI_{Ca}\cdot e^{\frac{Vm(t)-Vthresh}{S_{IV}}}$ | (5) |
| --- | --- |

where *V_thresh_* is the threshold of calcium current activation (-43 mV) and S_IV_ is the “slope factor” the depolarization generating an e-fold increase in I_Ca_ (6.6 mV). The amplitude of the calcium current therefore varied with the square of the radius of the terminal (see 2.2).

**2.4 Calcium impulse response:** The calcium transient in the synaptic terminal was calculated as the convolution of calcium influx and the “calcium impulse response” of the terminal (*Impulse_Ca_(t)*). The time-course of *Impulse_Ca_* was the product of an exponential rising phase, *f_rise_(t)* and exponential decay phase, *f_decay_(t)* as follows:

| $Ca\_impulse(t)=Ca_{free}\cdot f_{rise}(t)\cdot f_{decay}(t)$ | (6) |
| --- | --- |
| $Ca_{free}=peakI_{Ca}\cdot\frac{2zA}{Vk}$ | (7) |
| $f_{rise}(t)=1-e^{\frac{t}{\tau_{rise}(Ca)}}$ | (8) |
| $f_{decay}(t)=e^{\frac{t}{\tau_{decay}(Ca)}}$ | (9) |

Where *z* is the net charge of one electron (1.602*10^-19^ C), *A* is Avogadro’s number (6.022*10^-23^) and the factor of 2 accounts for divalence of calcium ions. *V* is terminal volume and *κ* is the buffer ratio (= 800), such that one in κ ions remains free. For simplicity, τ_rise(Ca)_ was 30 ms in all terminals. However, τ_decay(Ca)_ depended on radius as

| $\tau_{decay}=r\cdot\tau_{rad}$ | (10) |
| --- | --- |

where τ_rad_ = 755 ms µm^-1^, as measured experimentally (Figure 2J) . The peak amplitude of the calcium impulse therefore varied as the inverse of the Surface Area to Volume ratio (SVR), while τ_decay_ was proportional to radius.

Convolution of the calcium impulse response (2.4) with the calcium current (2.3) yielded changes in free calcium in the synaptic compartment.

**2.5 Dynamics of vesicle fusion:** Next we incorporated bulk calcium levels driving exocytosis through the ribbon complex[[4](#_ENREF_4), [5](#_ENREF_5)]. We assumed a separation of vesicles into three distinct pools[[9](#_ENREF_9)]. The readily releasable pool (RRP) relates to vesicles in direct contact with the cell membrane underneath the ribbon, while the intermediate pool (IP) represents vesicles that are tethered to the ribbon but not in direct contact with the membrane. The reserve pool (RP) relates to all vesicles not associated with ribbons. Numbers of vesicles in each pool and their relative rates of change were estimated from[[10](#_ENREF_10)], based on reported Ca^2+^-dependencies[[11-13](#_ENREF_11)]. Vesicle replenishment for each pool was modelled as:

| $\begin{matrix} \frac{dRP}{dt}=0 \\ \frac{dIP}{dt}=J_{RP\_to\_IP}-J_{IP\_to\_RRP} \\ \frac{dRRP}{dt}=J_{IP\_to\_RRP}-J_{exo} \end{matrix}$ | (11) |
| --- | --- |

where *J_RP_to_IP_* is the rate of refilling IP from RP, *J_IP_to_RRP_* is the rate of refilling of RRP from IP and *J_exo_* is the rate of exocytosis.

The size of RP depended on terminal volume, and was continuously replenished from the pool of exocytosed vesicles:

| $J_{Exo\_to\_RP}=F_{endo}\cdot Exo$ | (12) |
| --- | --- |

and

| $RP_{\max}=V\cdot D_{V}$ | (13) |
| --- | --- |

Where *J_Exo_to_RP_* is the rate of endocytosis, *F_Endo_* the fraction of exocytosed vesicles being endocytosed over time (0.1 s^-1^) and *Exo* the number of vesicle exocytosed from the RRP (see above). *RP_max_* is the maximal size of the reserve pool of vesicles, *V* is terminal volume and *D_V_* is vesicle density (=1050 vesicles µm^-3^, [[14](#_ENREF_14)])

Vesicles in the RP continuously refilled the IP independent of [Ca^2+^]:

| $J_{RP\_to\_IP}=J_{RP\_to\_RP\_\max}\cdot\frac{IP}{IP_{\max}}\cdot\frac{RP}{RP_{\max}}$ | (14) |
| --- | --- |

where J_IP_to_RP_ is the maximal rate of replenishment and IP_max_ is the maximal size of the intermediate pool. Vesicles in the IP translocate to the membrane as a Michaelis-Menten function of [Ca^2+^] with a K_d_ of 2 M to replenish the RRP:

| $J_{IP\_to\_RRP}=J_{IP\_to\_RRP\_\max}\cdot\frac{[Ca^{2+}]}{[Ca^{2+}]+K_{ip}}\cdot\frac{IP}{IP_{\max}}\cdot\frac{RRP}{RRP_{\max}}$ | (15) |
| --- | --- |

where J_IP_to_RRP_max_ is maximum rate of replenishment, K_ip_ is dissociation constant of the calcium sensor of the replenishment process and RRP_max_ is the maximal size of the readily releasable pool. Exocytosis occurred exclusively from the RRP, and was modeled to be linearly dependent on [Ca^2+^][[11-13](#_ENREF_11)].

- 1. **The impact of terminal size:** Four major aspects of the model depended on terminal dimensions.

1. The total calcium current (Calculation of calcium channel density, based on surface area. This influences the amplitude of the calcium current, according to peakI_Ca_ scale factor in equation 5, taken from Supplemental equation 3).
2. The time constant of decay of the calcium kernel, according to equation 10. τ_decay_ linearly depends on radius.
3. Calculation of ribbon number, based on surface area. The size of the RRP (docked vesicles) and IP (ribbon-attached vesicles) are both directly proportional to the number of ribbons.
4. Calculation of the size of the reserve pool of vesicles, which depends on the free volume of the terminal.

A list of all constants used in the model is shown in *Table S1*.

**3. A 3D Diffusion model of Ca^2+^.**

Calcium at ribbon synapses typically enters through clusters of calcium channels associated with the ribbon. To estimate the extent of any differences in the local calcium signal beneath the ribbon and bulk calcium we therefore set up a 3D model of calcium fluxes and diffusion using Virtual Cell (NIH: NRCAM), and evaluated at a resolution of 50 nm voxels. Input to the model was calcium current per active zone and the output was free calcium concentration at different distances to an active zone (Figure S3). Where appropriate, the model uses the same parameters as the numerical model (Table S2, cf. Table S1). The full 3D diffusion model is available in the public domain at <http://www.vcell.org/> under “Model for size paper”, shared username TomBaden.

**3.1 Calcium dynamics**

Calcium dynamics were modelled as 5 processes: influx, leak, buffering, extrusion and diffusion:

| $\frac{\partial[Ca^{2+}](t,x,y,z)}{\partial t}=D_{Ca}\nabla^{2}[Ca^{2+}](t,x,y,z)+J_{in}+J_{leak}-J_{buf}-J_{extr}$ | (16) |
| --- | --- |

where [Ca2+] is calcium concentration, D_Ca_ is the diffusion coefficient and J_in_, J_leak_, J_buf_ and J_extr_ are rates of calcium influx, leak, buffering and extrusion respectively (See Table S2).

i) Terminal geometry and calcium influx

Terminals were assumed to be spherical with different radii (1 and 5 μm). In bipolar cells, fast release of vesicles is driven primarily by calcium microdomains generated by clusters of calcium channels[[4](#_ENREF_4), [5](#_ENREF_5)]. We therefore modeled Ca^2+^ influx to occur exclusively at active zones, with each site of calcium influx passing a calcium current of 3 pA[[5](#_ENREF_5)] over a 150 x 150 nm patch of membrane (3 x 3 voxel-edges) [[4](#_ENREF_4)]. A 5 μm radius spherical compartment contained 50 equi-spaced active zones[[6](#_ENREF_6)], and numbers of active zones in compartments of other sizes were scaled to yield a constant density of ribbons per unit surface area. Accordingly, spherical compartments of radii of 1 and 5 μm comprised 2 and 50 active zones, respectively.

ii) Calcium leak and extrusion

Ca^2+^ leak and extrusion mechanisms across the cell membrane were distributed uniformly. All extrusion mechanisms collapsed into a single entity 10 μM[[8](#_ENREF_8), [15](#_ENREF_15)]:

| $Jextr=P_{\max}\cdot\frac{[Ca^{2+}]}{Kp+[Ca^{2+}]}$ | (17) |
| --- | --- |

where K_p_ is dissociation constant of the calcium pump (10 μM) and P_max_ the peak amplitude, chosen to yield a peak global Ca^2+^ concentration of 2 μM upon sustained activation of Ca^2+^ influx[[5](#_ENREF_5), [8](#_ENREF_8)]. Ca^2+^ leak was equal and opposite to extrusion at resting Ca^2+^ levels (i.e. [Ca^2+^]=50 nM).

iii) Calcium buffering

Two types of endogenous calcium buffers were used: a high affinity mobile buffer (‘fast’) and a lower affinity immobile buffer (‘slow’) [[5](#_ENREF_5), [16-18](#_ENREF_16)]. The fast mobile buffer was present at a concentration of 1.2 mM, had a forward rate-constant of calcium binding of 20 μM^-1^ s^-1^ and a diffusion coefficient of 20 μm s^-1^ [[5](#_ENREF_5), [16-18](#_ENREF_16)]. The slow fixed buffer (0.15 mM) had a forward rate-constant of calcium binding of 2 μM^-1^ s^-1^, with the diffusion coefficent set to zero. Dissociation constants of both buffers were 2.2 μM.

**3.2 Operation of the model**

To estimate calcium dynamics around sites of calcium influx the model was evaluated for the first 200 ms of continuous calcium channel activation with 10 microsecond time-steps. The average [Ca^2+^] at various distances from a site of calcium influx is shown on two different time-scales in Figure S3. The model predicts that in both small (red, 1 µm radius) and large (black, 5 µm radius) spherical compartments, calcium immediately adjacent to the site of influx (25 nm) is largely independent of compartment geometry within the first millisecond of channel opening (Figure S3A). However, at distances >25 nm or beyond the first millisecond, calcium levels are strongly influenced by terminal geometry (Figure S3A). Notably, at bipolar cell ribbon synapses, vesicle release is driven by calcium signals extending several hundreds of nanometers from the site of influx[[4](#_ENREF_4)]. Therefore the model suggests that bulk calcium can be readily used as an estimate of calcium driving release through the ribbon in bipolar cell synapses at time scales beyond a few milliseconds.

**4. The effect of changing L-type calcium channel threshold and calcium dependency of release: predictions of the model.** Two potential sources of non-linearity intrinsic to BC terminals may be (i) the voltage threshold of L-type calcium channels relative to Vrest, and (ii) the relation between calcium concentration and release rate. We explored the effects of changing both parameters in the one-compartment model, using the size and frequency dependency of release modulation to temporal contrast as metric to compare. To explore the effect of a change voltage threshold of calcium channels we set Vthresh 3 mV above (-40 mV) and below (-46 mV) the value used in the model (-43 mV). The most striking result of this manipulation was a change in the absolute amount of calcium entering the terminal upon stimulation (Figure S4A, left). However, the amplitude of calcium modulation fell with increasing radius for all conditions (Figure S4A, right). Qualitatively similar effects were predicted for the rate of release (Figure 4B). We conclude that changing the relative voltage of Vrest and Vthresh did not qualitatively influence the main conclusions drawn, namely that smaller terminals better encode faster frequency temporal contrast.

Next we tested the effect of calcium dependency of release. Although we used a linear relation, as measured at the ribbon synapse of photoreceptors [[13](#_ENREF_13)], it has also been suggested that the relation may be cooperative with a power of 3-4, as measured at conventional synapses [[19](#_ENREF_19)]. The model predicts that the modulation of release in response to a fluctuating stimulus would be significantly larger if Ca^2+^ ions acted cooperatively to trigger vesicle fusion. The impact of such a change in the calcium-dependence of release is shown in Figure S4 C,D. Here we used the model to explore the modulation of release in the terminals (r = 1 µm) for two different Ca^2+^-dependencies: linear (Hill coefficient h = 1) and cooperative (h = 3).

**5. Encoding of high frequency components using spikes**

Active voltage spikes in BC terminals may provide an important ingredient in boosting high frequency components in the BC output[[3](#_ENREF_3), [20-23](#_ENREF_20)]. Spikes in BCs typically exhibit very low rates – thereby sparsening the BC voltage response to a time-varying stimulus. As a result, the relatively long inter-spike intervals should allow calcium to decay back to near baseline levels following a spike. Postsynaptic pooling of multiple such stochastically spiking BCs should therefore allow to recover the linear component of the generator in the BC population output. To test this idea we included membrane noise and a spike threshold into the model and compared the predicted responses of graded and spiking synapses to a “chirp” stimulus (Figure S6, cf Figure 3). Addition of membrane noise (Brownian motion noise; sd = 1,4 mV) to the generator obtained from linear transformation of the stimulus with the same kernel used in Figure 3 (Figure S6A) yielded a prediction of graded membrane voltage (black, Figure S6B). Spike threshold was set to yield an average of 1.5 spikes per second, with Ʈ = 300 ms refractory period (red). Bulk calcium (Figure S6C) and release rates (Figure S6D) were calculated as before, with a hill dependence (h = 2) of release on calcium (see Figure S4). Average release rates predicted from 100 graded (black) and 100 spiking (red) BCs are shown in Figure S6E. As predicted (Figure 6H) the graded mechanism imposes a powerful low pass filter. However, the spiking mechanism boosts high frequency components and allows to recover the linear components of the generator.

**6. Supplemental references**

1. Gollisch, T., and Meister, M. (2010). Eye smarter than scientists believed: neural computations in circuits of the retina. Neuron *65*, 150-164.

2. Schnapf, J.L., Nunn, B.J., Meister, M., and Baylor, D.A. (1990). Visual transduction in cones of the monkey Macaca fascicularis. J Physiol *427*, 681-713.

3. Baden, T., Esposti, F., Nikolaev, A., and Lagnado, L. (2011). Spikes in retinal bipolar cells phase-lock to visual stimuli with millisecond precision. Curr Biol *21*, 1859-1869.

4. Beaumont, V., Llobet, A., and Lagnado, L. (2005). Expansion of calcium microdomains regulates fast exocytosis at a ribbon synapse. Proc Natl Acad Sci U S A *102*, 10700-10705.

5. Burrone, J., Neves, G., Gomis, A., Cooke, A., and Lagnado, L. (2002). Endogenous calcium buffers regulate fast exocytosis in the synaptic terminal of retinal bipolar cells. Neuron *33*, 101-112.

6. von Gersdorff, H., Vardi, E., Matthews, G., and Sterling, P. (1996). Evidence that vesicles on the synaptic ribbon of retinal bipolar neurons can be rapidly released. Neuron *16*, 1221-1227.

7. Holt, M., Cooke, A., Neef, A., and Lagnado, L. (2004). High mobility of vesicles supports continuous exocytosis at a ribbon synapse. Curr Biol *14*, 173-183.

8. Burrone, J., and Lagnado, L. (1997). Electrical resonance and Ca2+ influx in the synaptic terminal of depolarizing bipolar cells from the goldfish retina. J Physiol *505 ( Pt 3)*, 571-584.

9. Rizzoli, S.O., and Betz, W.J. (2005). Synaptic vesicle pools. Nat Rev Neurosci *6*, 57-69.

10. Neves, G., and Lagnado, L. (1999). The kinetics of exocytosis and endocytosis in the synaptic terminal of goldfish retinal bipolar cells. J Physiol *515 ( Pt 1)*, 181-202.

11. Jackman, S.L., Choi, S.Y., Thoreson, W.B., Rabl, K., Bartoletti, T.M., and Kramer, R.H. (2009). Role of the synaptic ribbon in transmitting the cone light response. Nat Neurosci *12*, 303-310.

12. Rouze, N.C., and Schwartz, E.A. (1998). Continuous and transient vesicle cycling at a ribbon synapse. J Neurosci *18*, 8614-8624.

13. Thoreson, W.B., Rabl, K., Townes-Anderson, E., and Heidelberger, R. (2004). A highly Ca2+-sensitive pool of vesicles contributes to linearity at the rod photoreceptor ribbon synapse. Neuron *42*, 595-605.

14. Odermatt, B., Nikolaev, A., and Lagnado, L. (2012). Encoding of luminance and contrast by linear and nonlinear synapses in the retina. Neuron *73*, 758-773.

15. Sala, F., and Hernandez-Cruz, A. (1990). Calcium diffusion modeling in a spherical neuron. Relevance of buffering properties. Biophys J *57*, 313-324.

16. McHugh, J.M., and Kenyon, J.L. (2004). An Excel-based model of Ca2+ diffusion and fura 2 measurements in a spherical cell. Am J Physiol Cell Physiol *286*, C342-348.

17. Schmidt, H., and Eilers, J. (2009). Spine neck geometry determines spino-dendritic cross-talk in the presence of mobile endogenous calcium binding proteins. J Comput Neurosci *27*, 229-243.

18. Wu, Y.C., Tucker, T., and Fettiplace, R. (1996). A theoretical study of calcium microdomains in turtle hair cells. Biophys J *71*, 2256-2275.

19. Heidelberger, R., Heinemann, C., Neher, E., and Matthews, G. (1994). Calcium dependence of the rate of exocytosis in a synaptic terminal. Nature *371*, 513-515.

20. Dreosti, E., Esposti, F., Baden, T., and Lagnado, L. (2011). In vivo evidence that retinal bipolar cells generate spikes modulated by light. Nature Neuroscience *14*, 951-952.

21. Protti, D.A., Flores-Herr, N., and von Gersdorff, H. (2000). Light evokes Ca2+ spikes in the axon terminal of a retinal bipolar cell. Neuron *25*, 215-227.

22. Saszik, S., and DeVries, S.H. (2012). A Mammalian Retinal Bipolar Cell Uses Both Graded Changes in Membrane Voltage and All-or-Nothing Na+ Spikes to Encode Light. The Journal of Neuroscience *32*, 297-307.

23. Baden, T., Euler, T., Weckstrom, M., and Lagnado, L. (2013). Spikes and ribbon synapses in early vision. Trends in neurosciences *36*, 480-488
